# A case report and literature review of rare intracranial and extracranial dual-lesion diffuse large B-cell lymphoma with heterogeneous subtypes (GCB type + ABC type)

**DOI:** 10.3389/fonc.2026.1802521

**Published:** 2026-04-01

**Authors:** Yadong Liu, Xuejuan Duan, Jinlong Liu, Chen Chen, Shaoning Yin, Gang Cheng, Meijian Yang, Wei Han, Na Li, Xianbo Zhang, Jing Zhao

**Affiliations:** 1 Department of Oncology, Hebei General Hospital, Shijiazhuang, China; 2 Department of Radiation Oncology, Fourth Hospital of Hebei Medical University, Shijiazhuang, China; 3 Graduate School, Hebei North University, Zhangjiakou, China; 4 Department of Pathology, Hebei General Hospital, Shijiazhuang, China; 5 Department of Hematology, Fourth Hospital of Hebei Medical University, Shijiazhuang, China

**Keywords:** activated B-cell type, diffuse large B-cell lymphoma, dual-subtype coexistence, germinal center B-cell type, individualized treatment, temporal muscle metastasis

## Abstract

Diffuse large B-cell lymphoma (DLBCL) is the most common subtype of malignant lymphoma in adults. According to the gene expression profile, it can be classified into germinal center B-cell (GCB) type and activated B-cell (ABC) type. Cases with coexistence of the two subtypes and cross-site involvement are extremely rare. Herein, we report a 65-year-old female patient who presented with blurred vision in the right eye. Imaging examinations revealed space-occupying lesions in the left thalamus, basal ganglia, deep temporal lobe and left temporal muscle. Pathological biopsy and immunohistochemistry confirmed that the intracranial lesion was ABC subtype DLBCL and the temporal muscle lesion was GCB subtype DLBCL. Fluorescence *in situ* hybridization (FISH) excluded c-MYC, Bcl-2 and Bcl-6 gene rearrangements. No involvement of other sites was detected by PET-CT and bone marrow biopsy. The patient initially received 2 cycles of rituximab combined with high-dose methotrexate chemotherapy. Efficacy evaluation showed stable disease (SD) of the intracranial lesion and complete response (CR) of the temporal muscle lesion. Subsequently, the regimen was adjusted to 6 cycles of cytarabine combined with temozolomide chemotherapy followed by radiotherapy for the intracranial lesion. Eventually, the intracranial lesion achieved partial response (PR) and the temporal muscle lesion maintained sustained CR. Up to the date of follow-up, the patient’s condition was stable without recurrence. Combined with literature review, this article discusses the possible mechanisms of the coexistence of dual-subtype DLBCL (clonal evolution or biclonal origin), the potential pathways of temporal muscle metastasis and the impact of subtype differences on treatment response, which provides clinical reference for the diagnosis and individualized treatment of such rare cases.

## Introduction

Diffuse large B-cell lymphoma (DLBCL) is one of the most heterogeneous subtypes of malignant lymphoma, accounting for approximately 30% of adult non-Hodgkin lymphoma (1). Based on gene expression profiling, DLBCL can be classified into two major subtypes: germinal center B-cell (GCB) type and activated B-cell (ABC) type. These subtypes exhibit significant differences in biological behavior, treatment sensitivity, and prognosis. The GCB subtype shows a higher response rate to rituximab-based chemotherapy with relatively favorable outcomes, whereas the ABC subtype is frequently associated with nuclear factor-κB (NF-κB) pathway activation, resulting in lower chemosensitivity, increased tendency for drug resistance, and poorer prognosis (2, 3). Clinically, DLBCL mostly presents as a single lesion or multisite involvement with homogeneous subtypes. However, the coexistence of both GCB and ABC subtypes in the same patient is extremely rare, and no literature has documented cases of cross-intracranial and extracranial dual-lesion involvement with heterogeneous subtypes (4, 5).

Primary central nervous system DLBCL (PCNSDLBCL) is a distinct subtype of DLBCL, accounting for 2%~3% of all DLBCL cases. It is highly aggressive, and treatment primarily consists of high-dose methotrexate-based chemotherapy combined with radiotherapy. Nevertheless, the incidence of extracranial metastasis is extremely low (only approximately 1%~2%), with metastatic sites mostly involving hematopoietic systems such as lymph nodes and bone marrow. No cases of involvement of extracranial muscular tissues (e.g., temporal muscle) have been reported (6, 7). As an extracranial skeletal muscle, the temporal muscle has an anatomical barrier with the intracranial vasculature. The pathways, mechanisms, and clinical characteristics of temporal muscle metastasis from PCNSDLBCL remain unclear, posing significant challenges to diagnosis and treatment.

In this study, we report a case of DLBCL with dual subtypes involving both the intracranial region and the temporal muscle. The diagnosis was confirmed through detailed clinical, imaging, pathological, and molecular biological examinations. The patient achieved favorable outcomes with an individualized treatment regimen. Combined with a review of previous literature, we discuss the possible mechanisms of dual-subtype coexistence, metastatic pathways, and treatment strategies, aiming to provide new insights and reference for the clinical diagnosis and management of such rare cases.

This case report was written in strict accordance with the CARE guidelines for case reports.

## Case presentation

**Figure 1:**
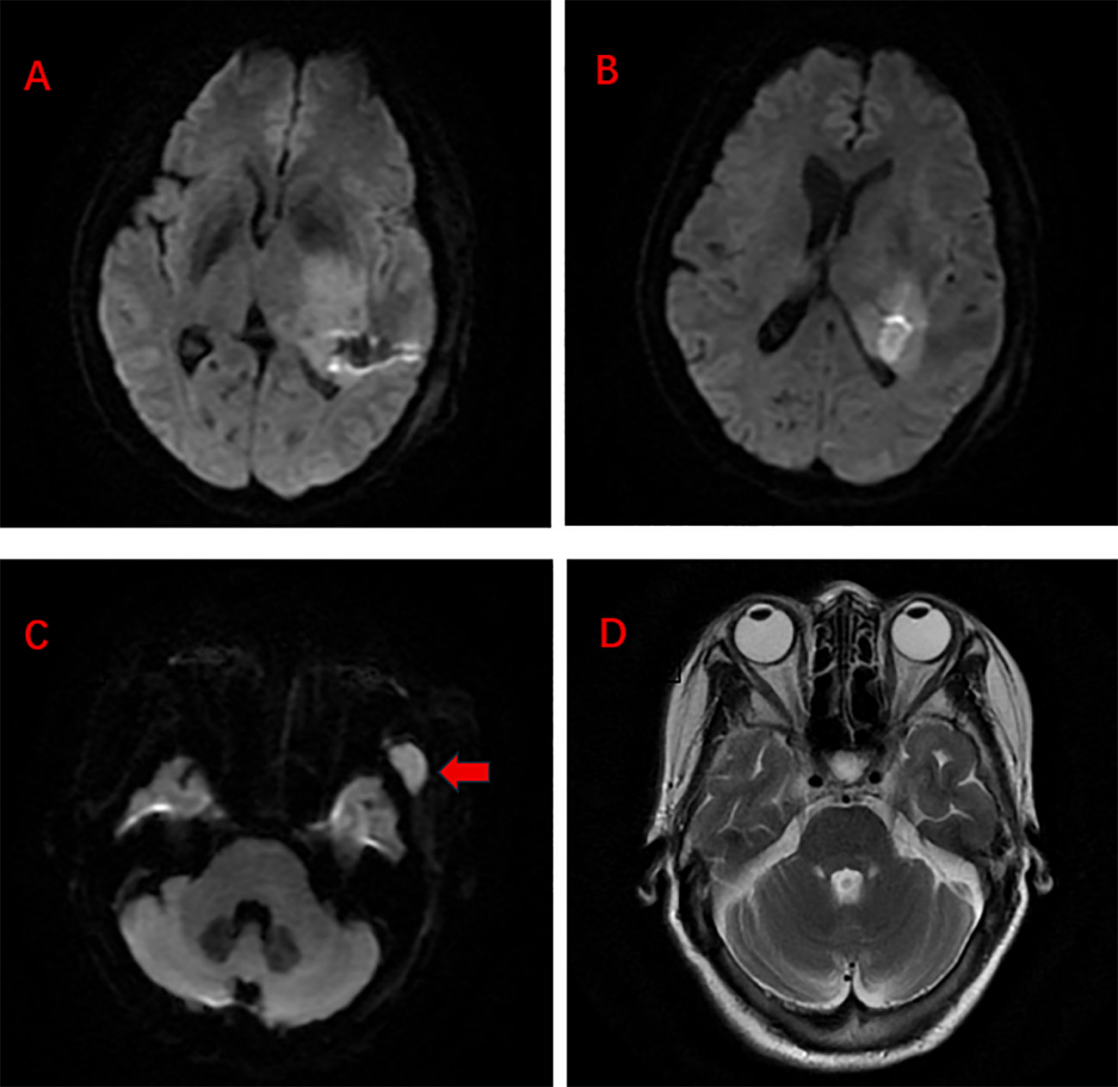
**(A, B)** Intracranial lymphoma lesions. **(C)** Temporal muscle metastases (red arrows). **(D)** Complete remission of temporal muscle metastases on reexamination.

A 65-year-old female patient presented to the outpatient department of our hospital in February 2025 with blurred vision in the right eye for 2 days. Visual acuity examination revealed a right temporal visual field defect, while the left visual field was normal. She had no accompanying headache, limb movement disorders, limb numbness, or dysphagia. Physical examination showed that the diameter of the left pupil was approximately 2.5 mm and that of the right pupil was about 3.0 mm. A right temporal visual field defect was observed, and the light reflex was sensitive. Ocular movement in all directions was normal without diplopia or nystagmus. Bilateral forehead wrinkles and nasolabial grooves were symmetrical; there was no deviation of the corner of the mouth when showing teeth, and the tongue protruded in the midline. Bilateral pharyngeal reflexes were present. Muscle strength of all limbs was Grade V, and muscle tone was normal. Cranial magnetic resonance imaging (MRI) indicated a high possibility of lymphoma in the left thalamus, left basal ganglia, deep left temporal lobe, and left temporal muscle (**Figures 1A–C**).

The patient had a healthy past medical history, with no chronic diseases such as hypertension, diabetes, or coronary heart disease; no history of infectious diseases like hepatitis or tuberculosis; no history of surgery, trauma, or blood transfusion; denied a history of food or drug allergies; had no history of long-term use of special medications before the onset of the disease; had no history of smoking or drinking; and had no family history of tumors or autoimmune diseases.

**Figure 2:**
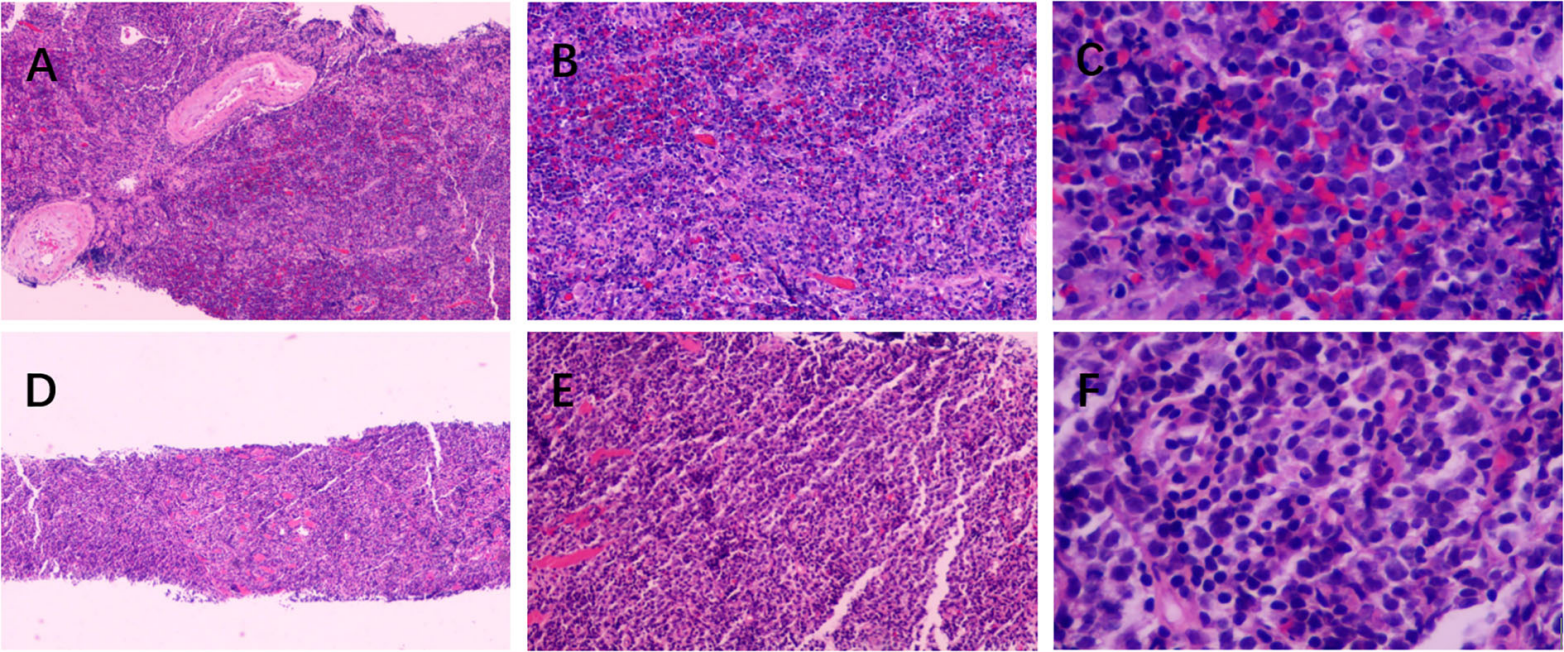
Pathological images. **(A–C)**. Pathological images of the intracranial lesion. **(D–F)**, Pathological images of Temporal Muscle Lesions. **(A, D)** (Hematoxylin and eosin (H&E) staining, ×40 magnification), **(B, E)** (Hematoxylin and eosin (H&E) staining, ×100 magnification), **(C, F)** (Hematoxylin and eosin (H&E) staining, ×400 magnification).

On March 3, 2025, the patient underwent stereotactic-guided craniotomy for biopsy under general anesthesia. Pathological examinationsuggested PCNSDLBCL of extra-germinal center activated B-cell origin (ABC type). Under the microscope, large, atypically shaped lymphoma cells are arranged in sheets. High-power microscopy shows pleomorphism of the large cells, with irregular nuclei, prominent nucleoli, and basophilic cytoplasm, which is consistent with the morphology of diffuse large B-cell lymphoma (**Figures 2A–C**). Molecular pathology showed EBER (-). Immunohistochemical staining (Supplementary Materials) revealed: CD3 (-), CD20 (+), CD79α (+), PAX-5 (+), CD21 (-), Ki-67 (>90% +), CD30 (-), P53 (scattered +), CD10 (-), MUM1 (80% +), CD5 (+), Cyclin D1 (-), ALK (-), c-MYC (approximately 20% +), Bcl-6 (60% +), Bcl-2 (>90% +), and GFAP (-).

On March 12, 2025, ultrasound-guided biopsy of the left temporal muscle mass was performed. Pathological results were consistent with diffuse large B-cell lymphoma of germinal center origin (GCB type), not otherwise specified (NOS). Biopsy revealed large sheets of transformed large B cells with prominent nucleoli and basophilic cytoplasm and a diffuse growth pattern. These represent typical morphological features of diffuse large B-cell lymphoma. (**Figures 2D–F**). Immunohistochemical staining (Supplementary Materials) showed: CD3 (-), CD20 (+), CD21 (-), Ki-67 (approximately 90% + in active areas), CD10 (+), MUM1 (70% +), c-MYC (approximately 15% +), Bcl-6 (60% +), and P53 (weak +). Fluorescence *in situ* hybridization (FISH) testing excluded c-MYC, Bcl-2, and Bcl-6 gene rearrangements. However, genetic analysis could not be performed due to the lack of residual tissue samples. Further positron emission tomography-computed tomography (PET-CT) examination showed no lesions in other sites, and bone marrow biopsy revealed no bone marrow involvement.

In this study, the germinal center B-cell (GCB) and non-GCB (activated B-cell, ABC) subtypes of DLBCL were classified strictly in accordance with the internationally recognized Hans algorithm, with the following interpretation criteria. Immunohistochemical staining results were independently evaluated by two senior hematopathologists using a double-blind method to ensure the objectivity and accuracy of the results. The Hans algorithm classification rules were implemented as follows: cases with positive CD10 expression were defined as the GCB subtype; cases with negative CD10 and negative Bcl-6 expression were categorized as the non-GCB (ABC) subtype; and for cases with negative CD10 and positive Bcl-6 expression, the GCB subtype was assigned if MUM1 was negative and the non-GCB (ABC) subtype if MUM1 was positive. For the present case, immunohistochemical analysis of the intracranial lesion revealed negative CD10, positive Bcl-6 and positive MUM1 expression, which conformed to the diagnostic criteria for the non-GCB (ABC) subtype per the Hans algorithm; in contrast, the temporal muscle lesion exhibited positive CD10 expression, consistent with the definition of the GCB subtype based on the same algorithm.

In this case, a systematic differential diagnosis was conducted based on clinical phenotypes, imaging features, and laboratory test results: High-grade gliomas typically present with ring enhancement of the lesion accompanied by significant peritumoral edema. However, the lesion in this case showed uniform enhancement without obvious necrosis, so this diagnosis was ruled out. Intracranial metastases usually have a history of systemic primary tumors, with lesions preferentially occurring at the junction of cerebral cortex and medulla, often accompanied by severe peritumoral edema. In this case, no extracranial primary tumor lesions were found by whole-body PET-CT, so this diagnosis was also excluded. Patients with central nervous system infections mostly present with infection-related toxic symptoms such as fever, headache, and meningeal irritation signs, and blood routine and cerebrospinal fluid examinations may show infection-related abnormal indicators, with abscess wall formation possible in the lesions. The patient in this case had no such typical manifestations, and relevant laboratory tests were normal, thus excluding this diagnosis. The diagnosis of this case followed a complete and traceable full-chain logic: First, the core sign of right temporal visual field defect was identified through the patient’s main complaint and physical examination. Cranial MRI indicated space-occupying lesions in the left thalamus, basal ganglia, deep temporal lobe, and left temporal muscle, highly suggesting the possibility of lymphoma. Subsequently, stereotactic-guided biopsy was performed on the intracranial lesions, and ultrasound-guided biopsy on the temporal muscle lesions in sequence. Combined with hematoxylin-eosin (HE) staining, immunohistochemistry, and FISH detection, the pathological typing and molecular characteristics of the lesions were clarified. Finally, whole-body PET-CT and bone marrow biopsy excluded involvement of other parts of the body, completing the final diagnosis of this case.

The patient received 2 cycles of rituximab combined with high-dose methotrexate chemotherapy on March 12 and March 31, 2025. Repeat cranial MRI in April 2025 showed lymphoma lesions in the left thalamus, left basal ganglia, deep left temporal lobe, and left cerebral peduncle. The lesion in the left cerebral peduncle showed no significant change compared with the previous examination (February 26, 2025), while the other lesions were significantly reduced. Efficacy evaluation indicated SD of the intracranial lesions and CR of the left temporal muscle lesion (**Figure 1D**). Subsequently, the treatment regimen was adjusted to 6 cycles of cytarabine combined with temozolomide chemotherapy, followed by radiotherapy for the intracranial lesions. Post-treatment imaging re-examination showed PR of the intracranial lesions and sustained CR of the temporal muscle metastatic lesion, with no recurrence. The last follow-up was in February 2026. During 2 cycles of R-HD-MTX chemotherapy, the patient developed grade I myelosuppression, which rapidly resolved after symptomatic and supportive treatment, with no other adverse events such as liver or kidney function damage. During 6 cycles of cytarabine combined with temozolomide chemotherapy, the patient experienced grade III myelosuppression once, which improved after symptomatic treatment, with no other adverse events. The patient strictly followed the doctor’s advice throughout the entire course to complete all chemotherapy cycles and intracranial radiotherapy, with no chemotherapy delays, dose reductions, or treatment interruptions, showing good treatment compliance. Up to the present, the patient’s condition remains stable and her physical status is good.

Informed consent was obtained from the patient for this publication. The investigation was conducted in accordance with the Declaration of Helsinki (1975). This study was also approved by the Ethics Committee of Hebei General Hospital (No. 2025-339).

## Discussion

Diffuse large B-cell lymphoma (DLBCL) is the most common subtype of malignant lymphoma, accounting for 30% of adult lymphoma cases (8). Since 2000, gene expression profiling studies have confirmed that DLBCL has two major subtypes: germinal center B-cell (GCB) type and activated B-cell (ABC) type (9). In routine clinical practice, pathologists usually classify these two DLBCL subtypes using the Hans algorithm, which distinguishes DLBCL into GCB or non-GCB type through immunohistochemical detection of CD10, BCL6, and MUM1. This subtyping directly affects prognosis and treatment regimen selection (10). Clinically, the coexistence of DLBCL with other lymphomas or tumors has been reported, but cases of coexisting dual DLBCL subtypes are relatively rare.

A previous study reported a case of DLBCL with mixed GCB type (CD10+, BCL6+, MUM1-) and non-GCB type (CD10-, BCL6+, MUM1+) components in the same lymph node (11). Genetic testing indicated clonal relatedness between the two components; in the non-GCB region, immunohistochemistry showed BCL2 positivity, and FISH detected BCL2 amplification. However, neither of these findings was observed in the GCB lesion. These results suggest that GCB-type DLBCL may progress to non-GCB-type DLBCL with BCL2 amplification.

Malignant lymphoma may sometimes undergo significant morphological and/or phenotypic changes. Low-grade B-cell lymphoma can occasionally experience dramatic transformations in morphology and immunophenotype. In several previous studies, 9% and 16% of DLBCL patients transformed from follicular lymphoma were of non-GCB type, mainly due to loss of CD10 expression and retention or acquisition of MUM1 expression (12). According to previous clonal evolution studies, transformed specimens are usually composed of clones that are rare or absent in the initial follicular lymphoma specimens, which is consistent with the significant clonal expansion that dominates the transformed specimens (13).

The pathogenesis of primary central nervous system lymphoma remains unclear. Some scholars have pointed out that chronic inflammation caused by previous head trauma may be the pathological basis for the development of this disease. The possible mechanisms of extracranial metastasis of central nervous system lymphoma include anatomical barrier destruction, hematogenous dissemination, and lymphatic system pathways (14). Studies have shown that extracranial metastatic GBM often exhibits specific gene mutation patterns, including isocitrate dehydrogenase (IDH) wild type, TERT promoter mutation, PTEN mutation, TP53 mutation, and RB1 mutation. These genetic changes may enhance the invasiveness and metastatic ability of tumor cells. Epithelial-mesenchymal transition-related genes may play a key role in this process (15). Epithelial-mesenchymal transition also plays a key role in extracranial metastasis. Studies have shown that activation of the TGF-β pathway is an important inducer of EMT, which can enhance the invasiveness and metastatic ability of tumor cells. In addition, enhanced intercellular adhesion is also considered one of the important mechanisms of lymphatic metastasis (16). These molecular changes may work synergistically to promote tumor cells to cross the blood-brain barrier and spread to extracranial tissues.

The coexistence of DLBCL lesions with both GCB and ABC subtypes in the same patient may involve two possible mechanisms: 1) Clonal evolution of tumor cells: The initial tumor clone undergoes changes in gene expression profile during proliferation and metastasis, leading to subtype switching; 2) Biclonal origin: Two independent DLBCL clones occur simultaneously in the patient, presenting distinct subtype characteristics (17, 18). Previous literature has reported cases of distant metastasis of intracranial lymphoma (19), but no cases of intracranial lesions combined with temporal muscle metastasis have been documented. As an extracranial muscular tissue, extracranial soft tissue metastasis of PCNSDLBCL is extremely rare, and its metastatic pathway remains incompletely understood. The possible mechanisms are speculated as follows: During the progression of intracranial lymphoma, clonal evolution occurs, and tumor cells spread to extracranial soft tissues through the cerebrospinal fluid circulation via skull base foramina; or the tumor invades the skull and directly infiltrates the surrounding muscular tissues; it may also be related to the high invasiveness of tumor cells and abnormal expression of adhesion molecules, enabling them to break through the blood-brain barrier and intracranial confinement to develop distant metastasis. Pathological examination showed that the intracranial lesion was of extra-germinal center activated B-cell origin, while the temporal muscle metastatic lesion was of germinal center origin. This difference in origin suggests that the tumor may have clonal heterogeneity, a feature that may affect the biological behavior of the tumor and its responsiveness to treatment, which requires further verification in subsequent studies. In addition, the biclonal origin of the intracranial and temporal muscle lesions can also explain the phenomenon reported in this case. If in the future, immunoglobulin clonality analysis and somatic mutation profile comparison of the two lesions can be performed via NGS, it will ultimately clarify the clonal origin relationship between the two lesions and provide more direct evidence for the pathogenesis of double-subtype DLBCL.

Previous literature has reported that GCB and ABC subtypes differ in prognosis and treatment sensitivity: the GCB subtype has a relatively higher response rate to rituximab-based chemotherapy and a better prognosis; the ABC subtype is often associated with nuclear factor-κB (NF-κB) pathway activation, showing lower sensitivity to rituximab-based chemotherapy, poorer prognosis, and higher tendency for drug resistance (20, 21). The results of the multivariate analysis and Kaplan-Meier survival curve comparison from the study conducted by Rizzuto I showed that the immunohistochemical subtype and the degree of contrast enhancement in MRI examinations are the most significant indicators affecting overall survival. Among them, patients with the germinal center B-cell type had a better prognosis and a longer overall survival (16 months), while patients with the non-germinal center B-cell type had a poorer prognosis and a shorter overall survival (8 months). Compared with cases with low enhancement values corresponding to the non-germinal center B-cell type, cases with high MRI enhancement values corresponding to the germinal center B-cell type had a longer survival time (22).

The present case also confirms these views: the intracranial lesion was of ABC subtype and showed no significant improvement after 2 cycles of rituximab combined with methotrexate chemotherapy, while the temporal muscle lesion (GCB type) achieved CR, which is consistent with previous literature reports. The coexistence of dual subtypes means that the treatment regimen cannot be formulated based solely on the subtype of one lesion; instead, the treatment needs of both subtypes must be considered, and the necessity of intensive treatment regimens should be evaluated. High-dose methotrexate is a cornerstone drug in the treatment of PCNSDLBCL. It has a high penetration rate through the blood-brain barrier, with the drug concentration in cerebrospinal fluid reaching more than 50% of that in plasma, making it the first-choice chemotherapy drug for intracranial DLBCL. Rituximab can achieve effective intracranial drug concentrations in PCNSDLBCL patients whose blood-brain barrier is damaged by tumors, and at the same time, it has clear anti-tumor activity against extracranial GCB subtype lesions. This regimen is the first-line standard treatment for PCNSDLBCL. Efficacy evaluation indicates that the intracranial ABC subtype lesions are in SD, and the extracranial GCB subtype lesions have achieved CR. Considering that the ABC subtype is less sensitive to the R-HD-MTX regimen, while cytarabine has no cross-resistance with methotrexate, has excellent blood-brain barrier penetration, and its combination with temozolomide has synergistic anti-tumor activity against PCNSDLBCL. Meanwhile, temozolomide has a clear anti-tumor effect on ABC subtype DLBCL with activated NF-κB pathway, which can cover the treatment needs of both intracranial ABC subtype and extracranial GCB subtype lesions, and fully take into account the biological heterogeneity of the dual-subtype disease (23). DLBCL involving both the intracranial region and temporal muscle is rare, and there is currently no unified treatment standard. The stable condition and long-term recurrence-free survival of the patient in this case after individualized treatment suggest that early definitive diagnosis, timely initiation of targeted chemotherapy, and prompt adjustment of treatment strategies based on efficacy may improve patient prognosis.

This study has significant clinical value along with certain research limitations. Its strengths are mainly reflected in three aspects: first, this case is the first internationally reported case of diffuse large B-cell lymphoma with two lesions crossing intracranial and extracranial anatomical sites and heterogeneous coexistence of GCB and ABC subtypes. Meanwhile, it is the first discovery of a rare clinical phenotype where primary central nervous system diffuse large B-cell lymphoma metastasizes to the temporal muscle, filling the gap in case reports in this field; second, this study completely records the differential therapeutic responses of lesions of different subtypes to chemotherapy regimens, providing direct clinical practice references for formulating individualized treatment plans for double-subtype diffuse large B-cell lymphoma; third, combining case analysis and literature review, this study systematically discusses the pathogenesis of double-subtype diffuse large B-cell lymphoma and the potential pathways of extracranial muscle metastasis of primary central nervous system diffuse large B-cell lymphoma, offering new ideas and directions for subsequent related mechanism research. The limitations of this study cannot be ignored: due to insufficient remaining tissue samples from the biopsy, gene expression profiling and next-generation sequencing tests could not be carried out, making it impossible to clarify the clonal origin relationship between the two lesions through clonal tracing analysis. Only the immunohistochemical Hans algorithm was used for subtype classification, which has certain methodological limitations; this study is a single-case report, and the relevant clinical and mechanistic conclusions still need to be further verified by more large-sample, multi-center case series studies; at the same time, this study could not verify the correlation between the molecular phenotypic differences of the two lesions and tumor metastatic characteristics through *in vitro* experiments, and the discussion on related mechanisms remains at the hypothetical level, which needs to be confirmed by subsequent targeted basic experimental studies.

## Conclusion

This case reports the diagnosis and treatment process of a rare case of diffuse large B-cell lymphoma involving both the intracranial region and temporal muscle with heterogeneous subtypes. Favorable clinical outcomes were achieved through initial treatment with rituximab combined with high-dose methotrexate followed by cytarabine combined with temozolomide. This case provides clinical experience for the possible patterns and treatment options of lymphomas with different subtypes in the same patient. In the future, more cases need to be accumulated, and large-sample clinical studies should be conducted to further clarify the pathogenesis, optimal treatment regimens, and prognostic factors of such rare cases, thereby improving the level of clinical diagnosis and treatment.

## Data Availability

The original contributions presented in the study are included in the article/**Supplementary Material**. Further inquiries can be directed to the corresponding author.
