# Supplementary material for: A case report and literature review of rare intracranial and extracranial dual-lesion diffuse large B-cell lymphoma with heterogeneous subtypes (GCB type + ABC type)
Source: Front Oncol. 2026 Apr 1;16:1802521. doi: 10.3389/fonc.2026.1802521 (PMC13079187; doi:10.3389/fonc.2026.1802521)

Figure 1: Immunohistochemical images of the intracranial lesion

ALK ×100


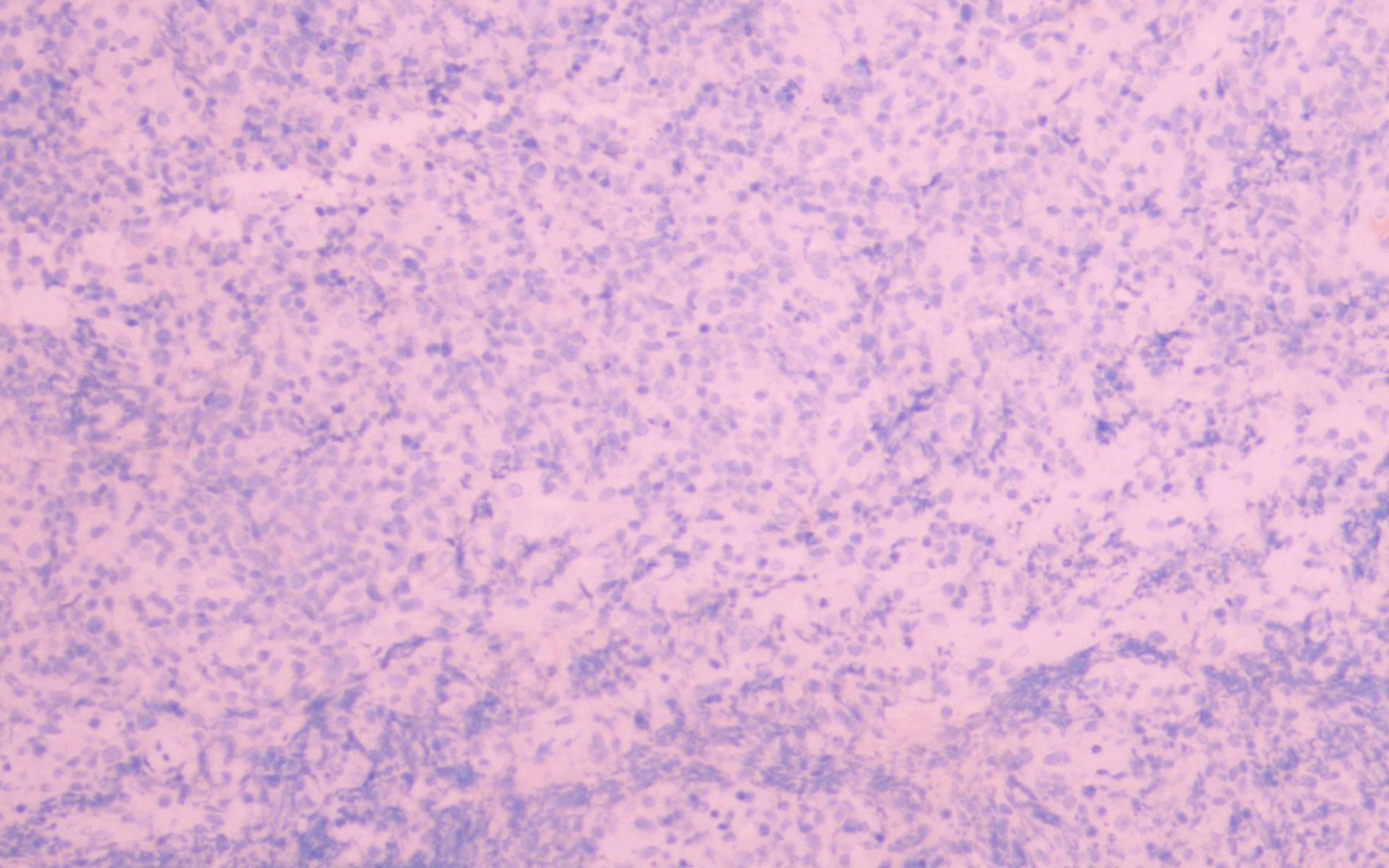


Bcl-2 ×100
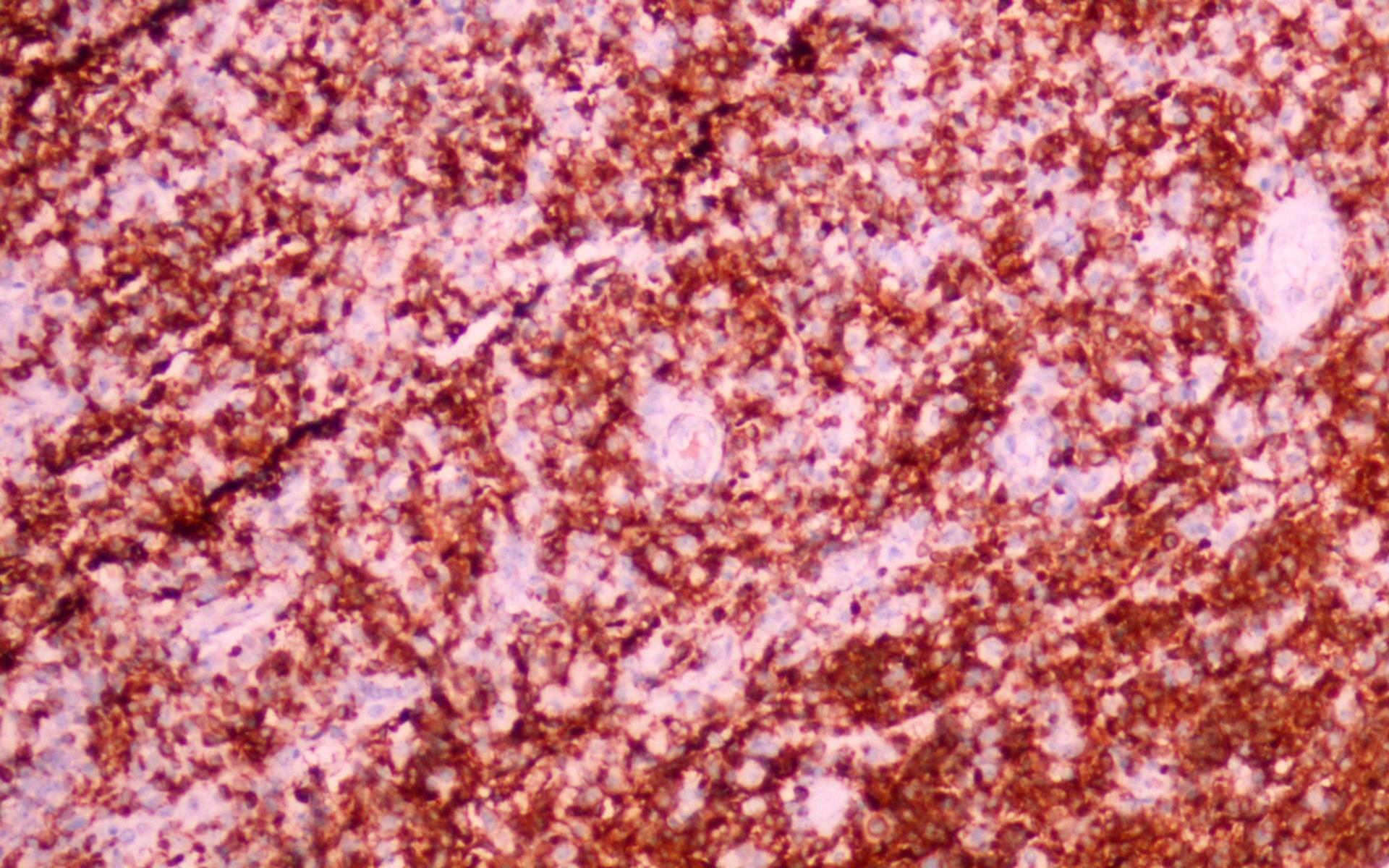


Bcl-6 ×100
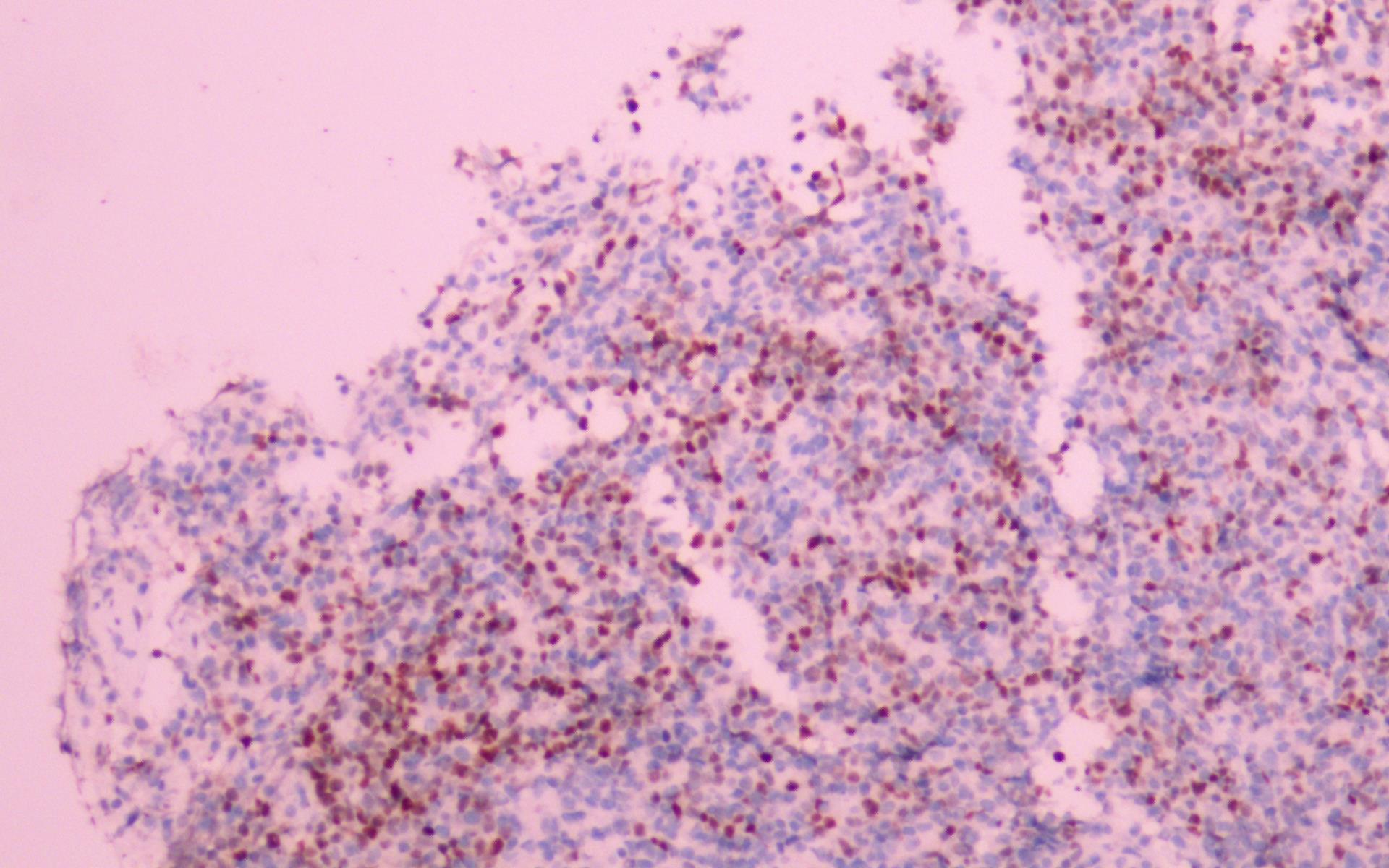


CD3 ×100
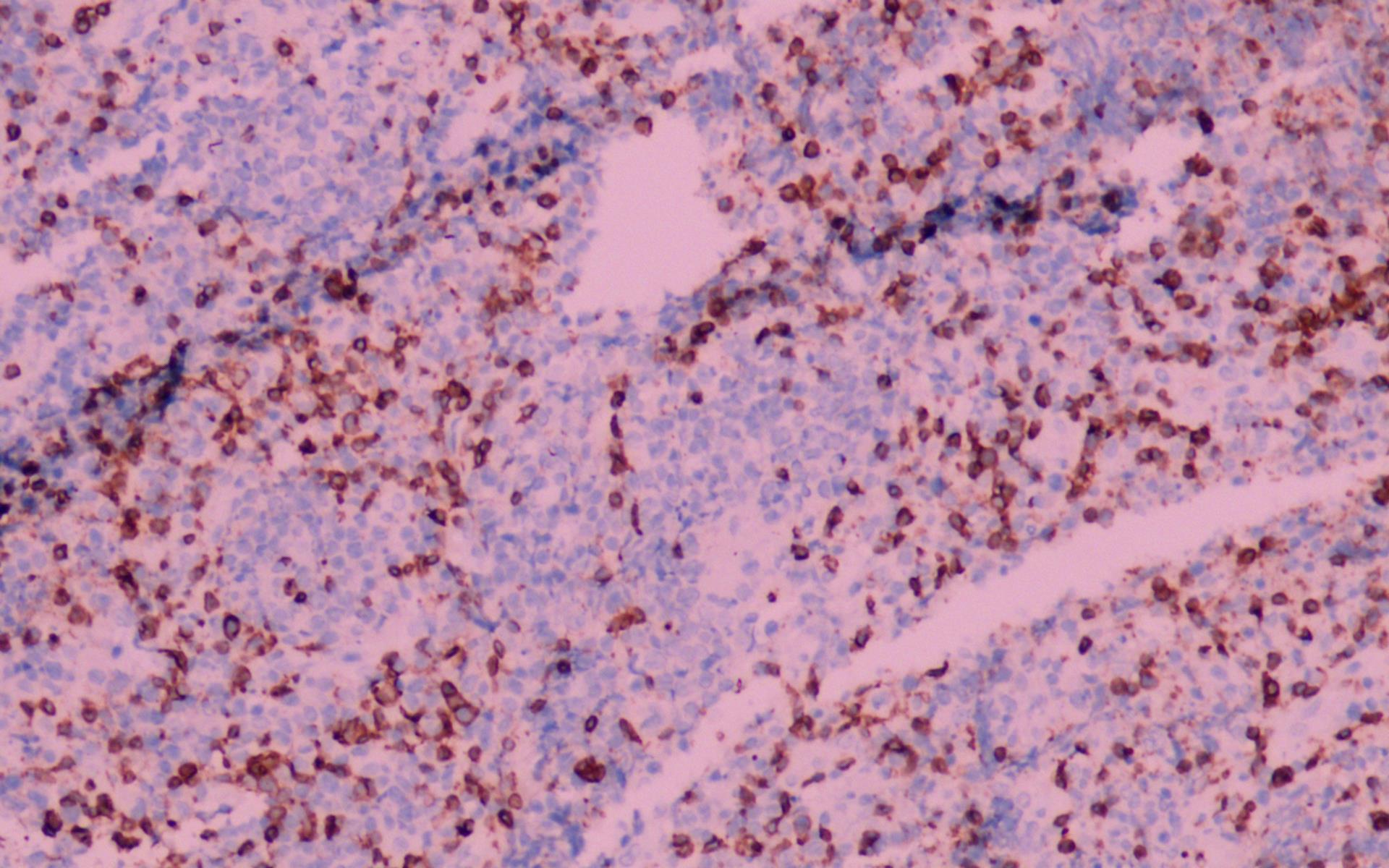


CD5 ×100
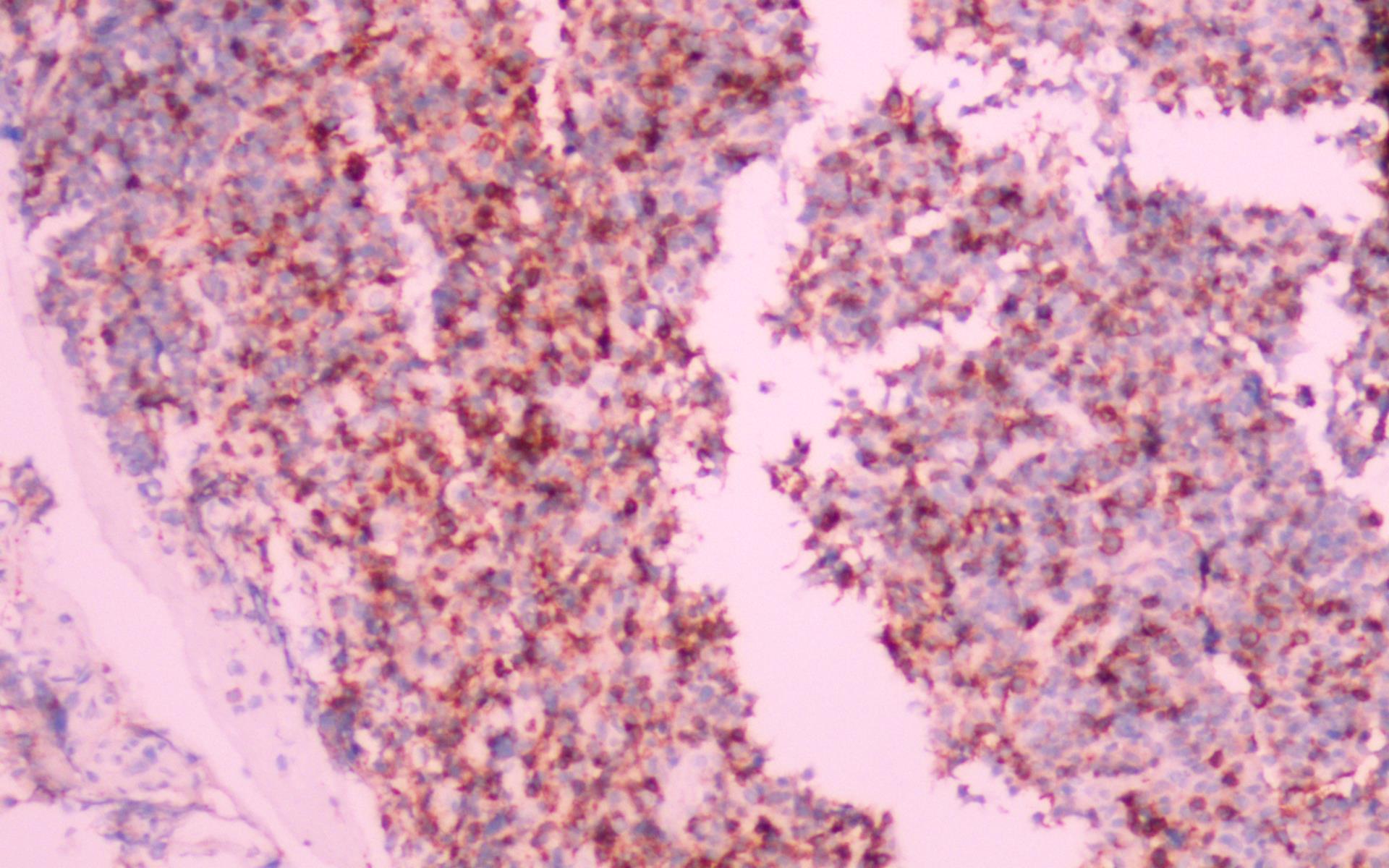
CD10 ×100
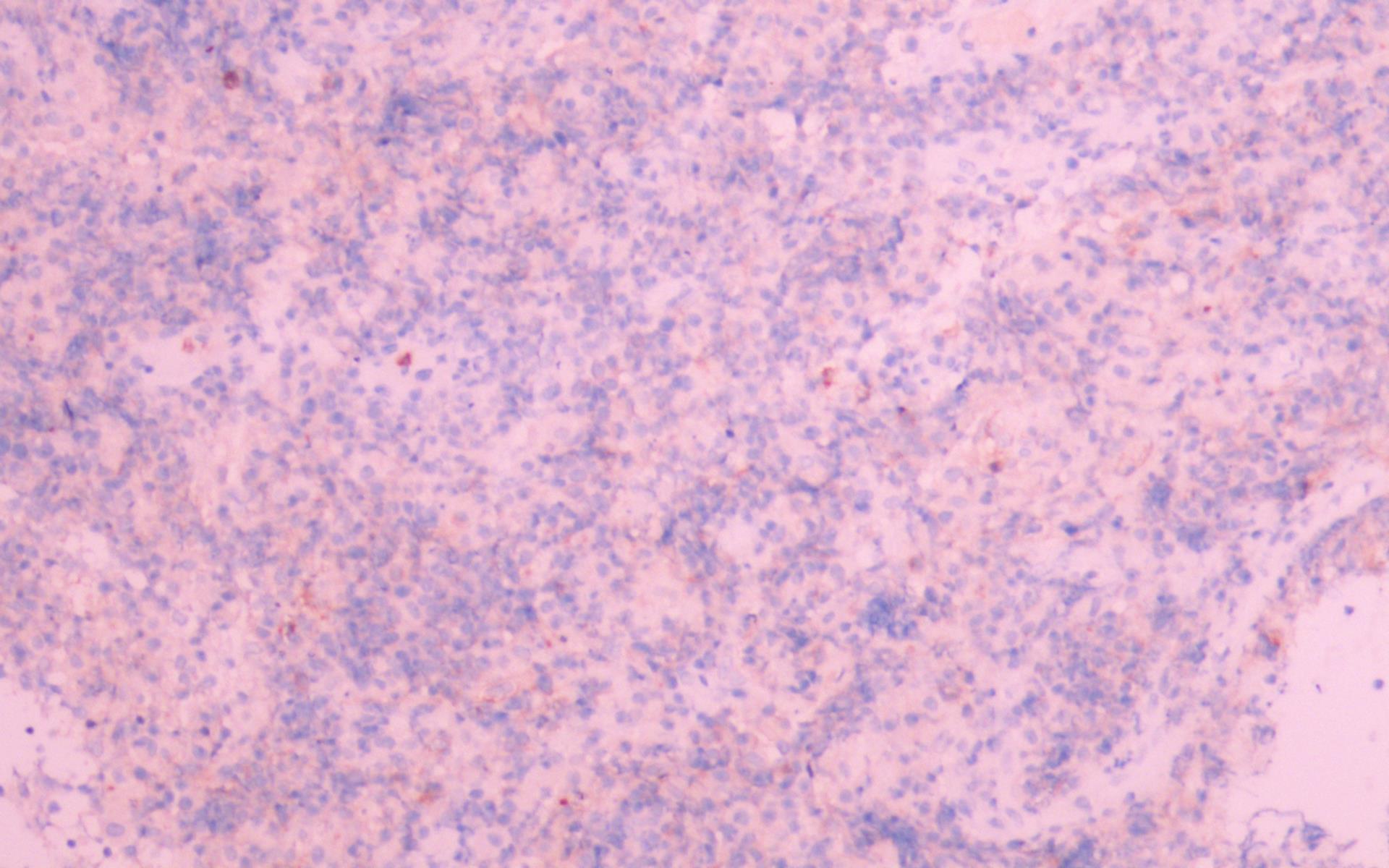


CD20 ×100
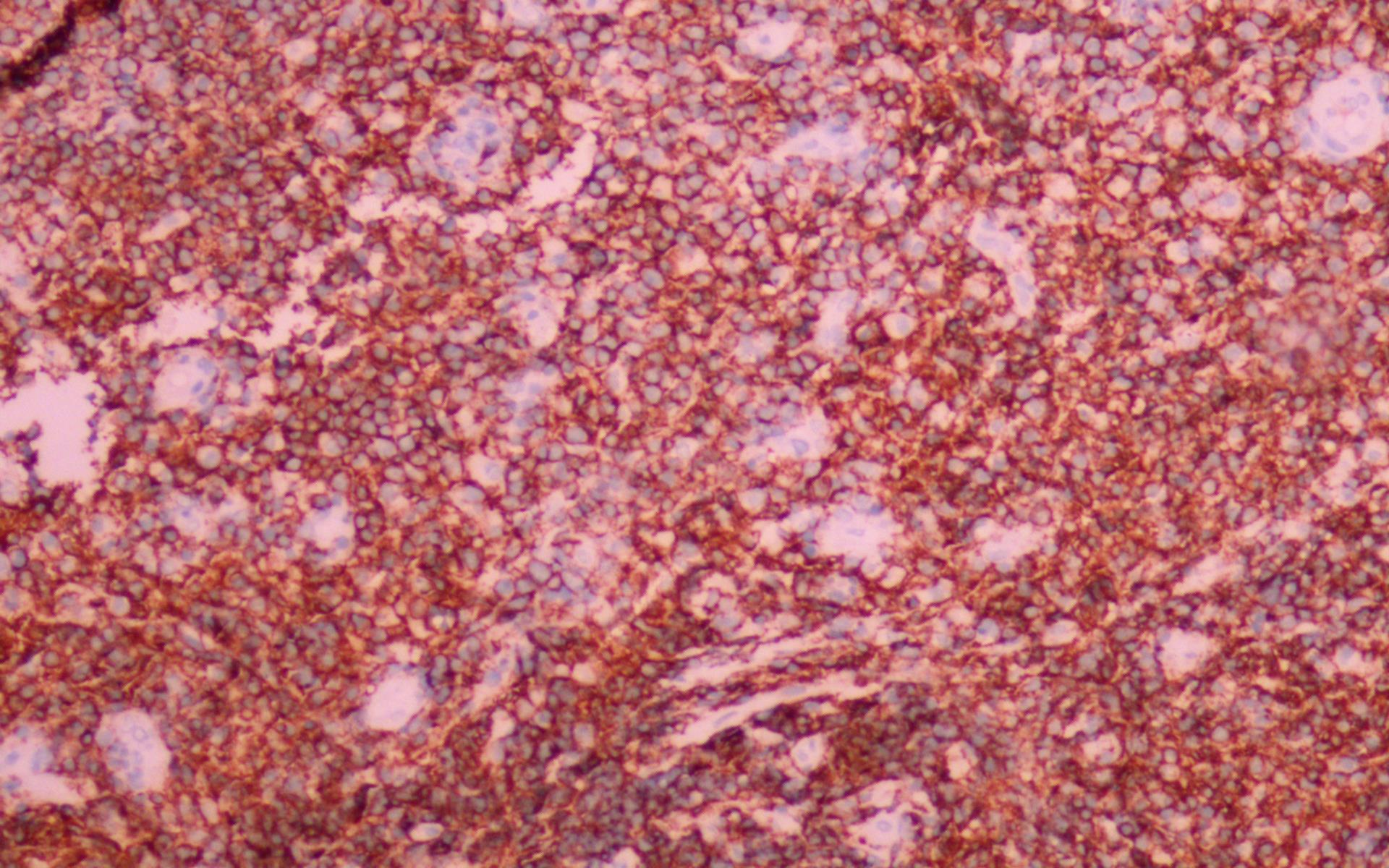
CD21 ×100
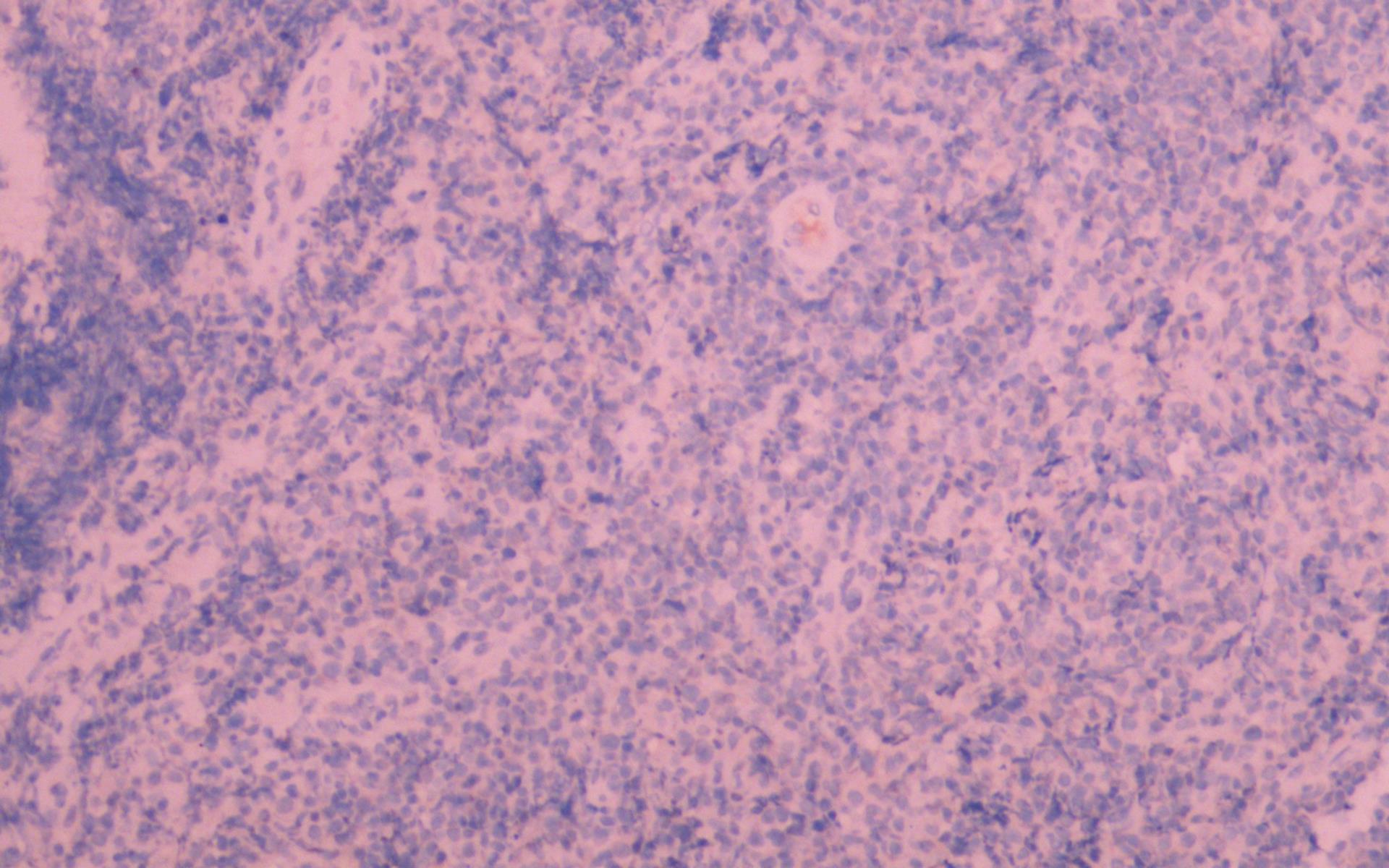


CD30 ×100
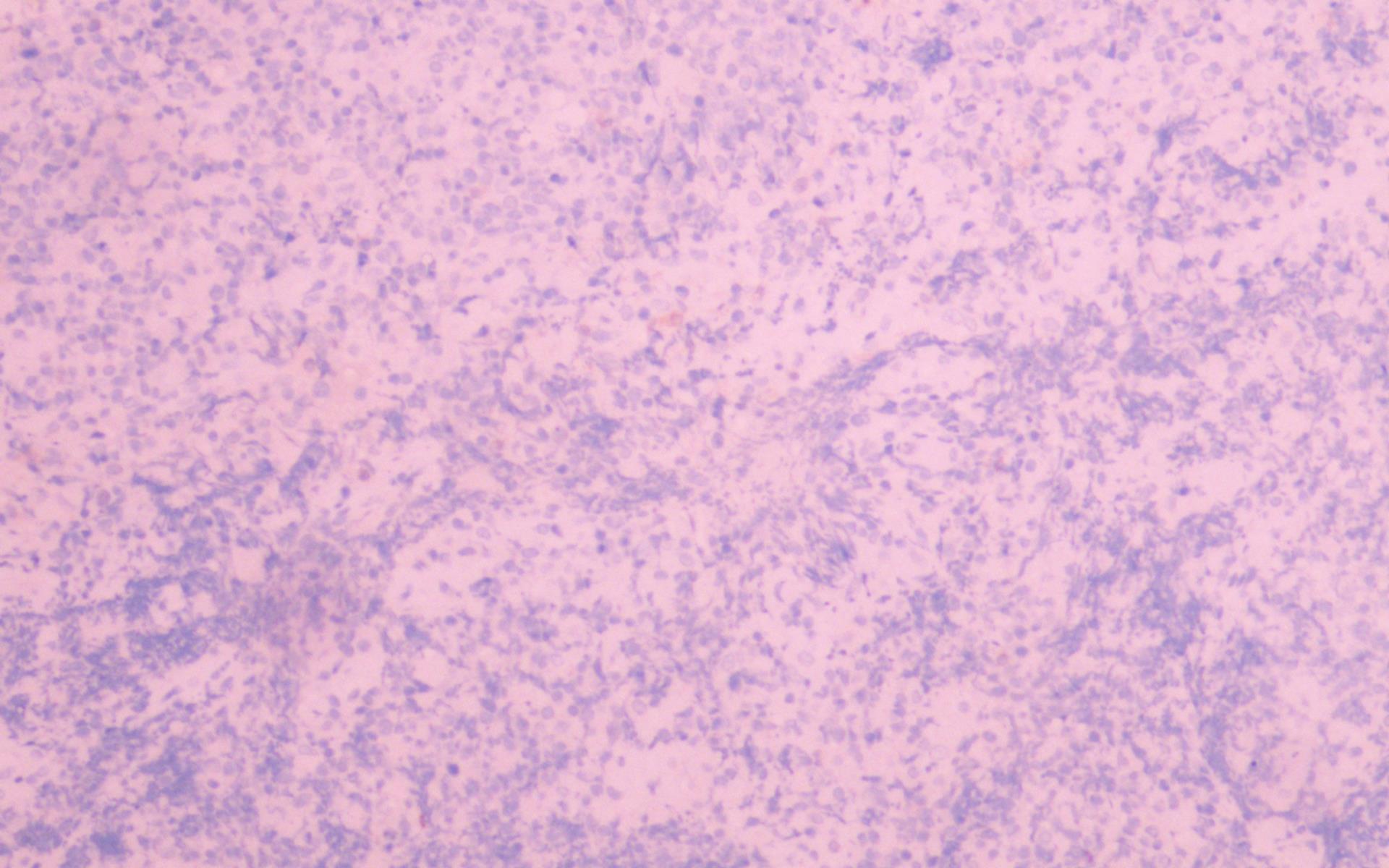
CD79α ×100
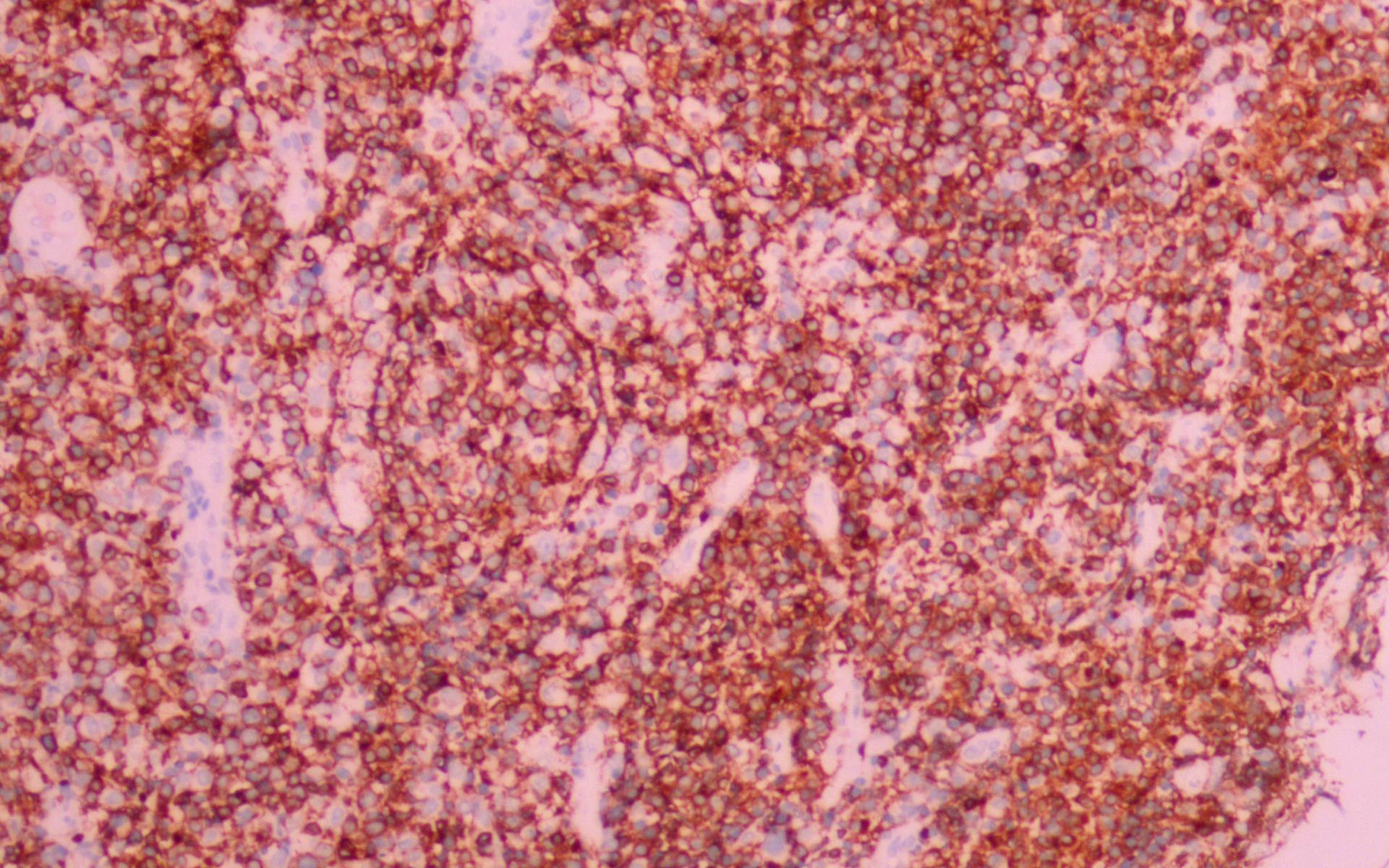


cMYC ×100
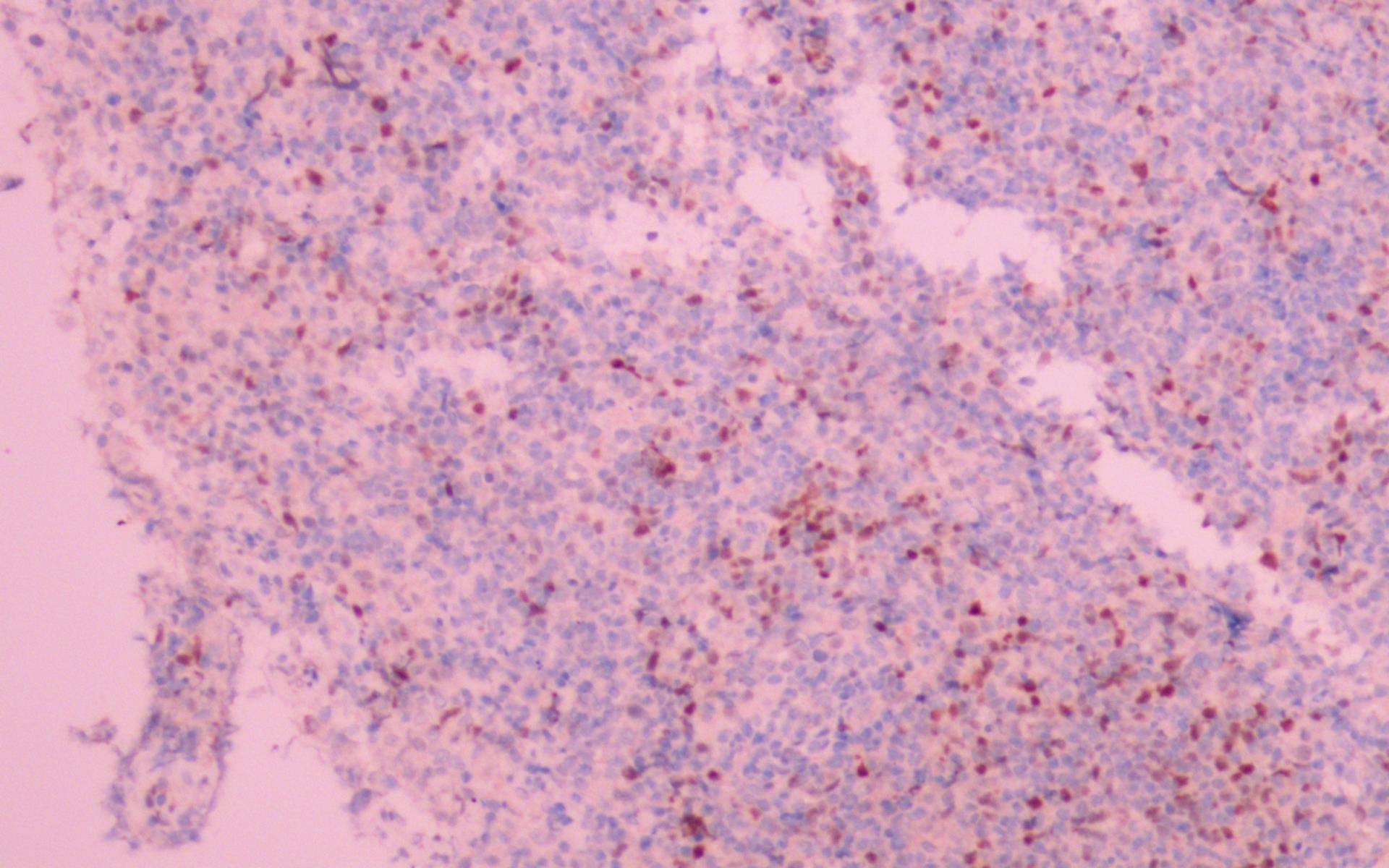
Cyclin D1 ×100
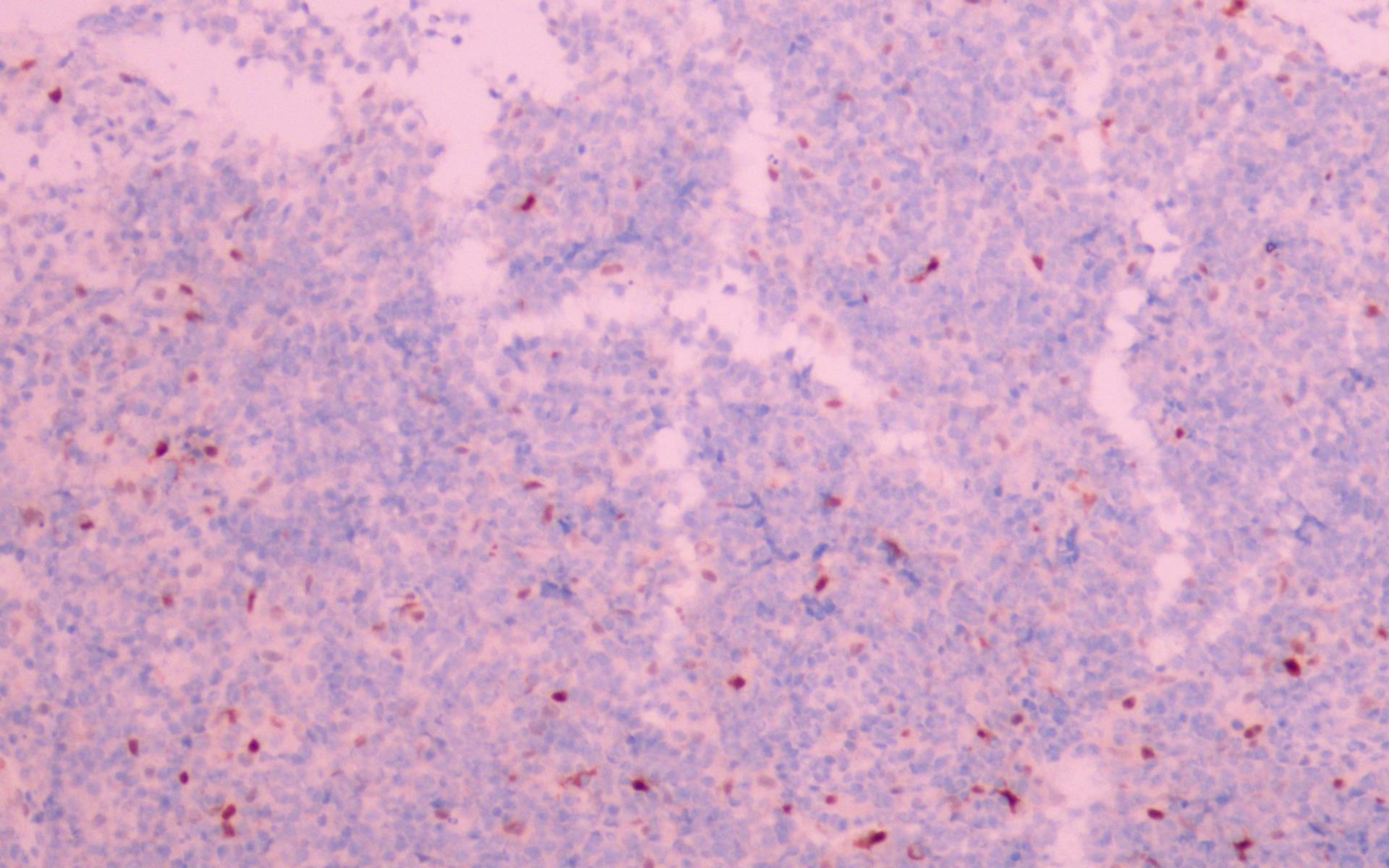


EBER ×100
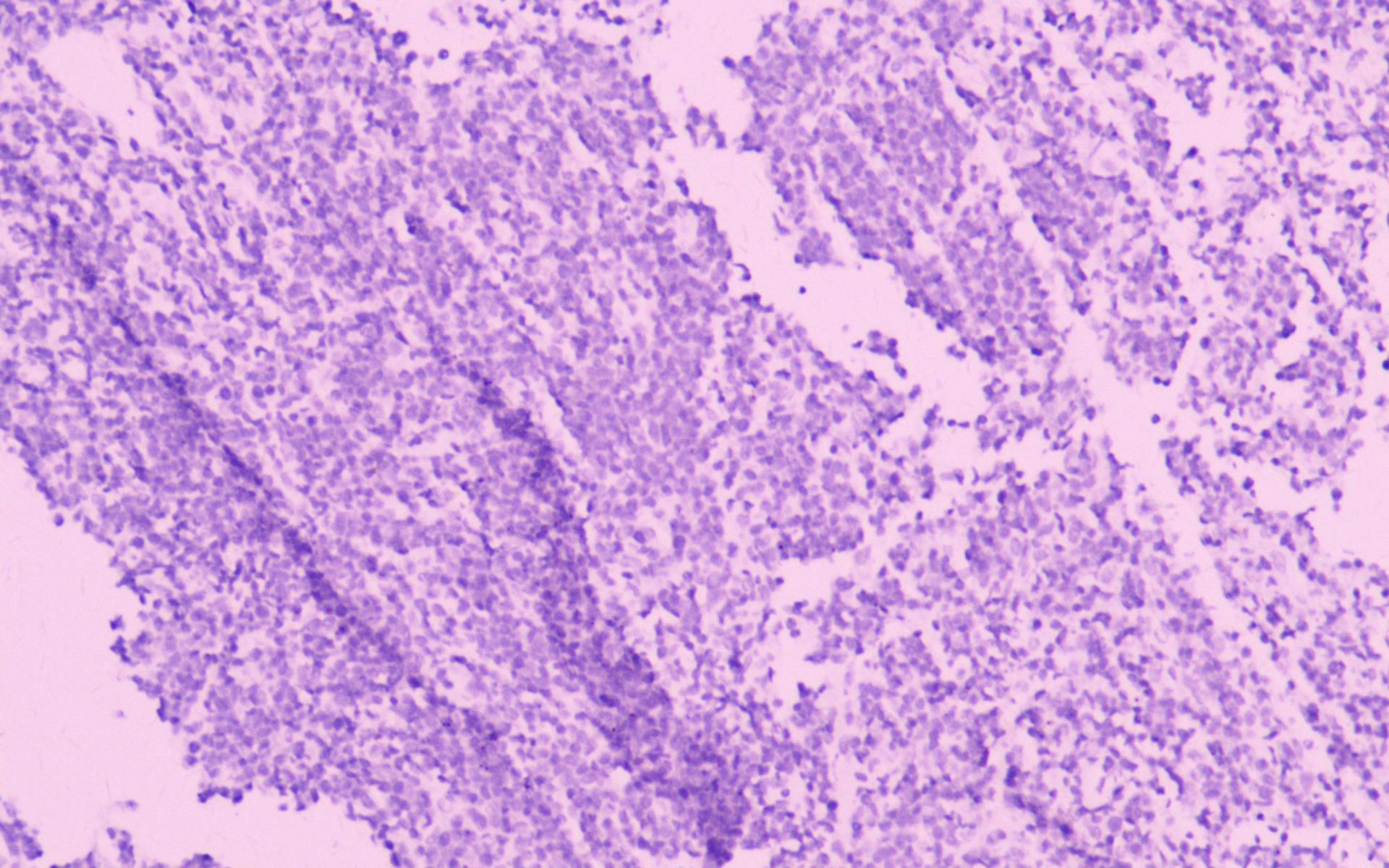
GFAP ×100
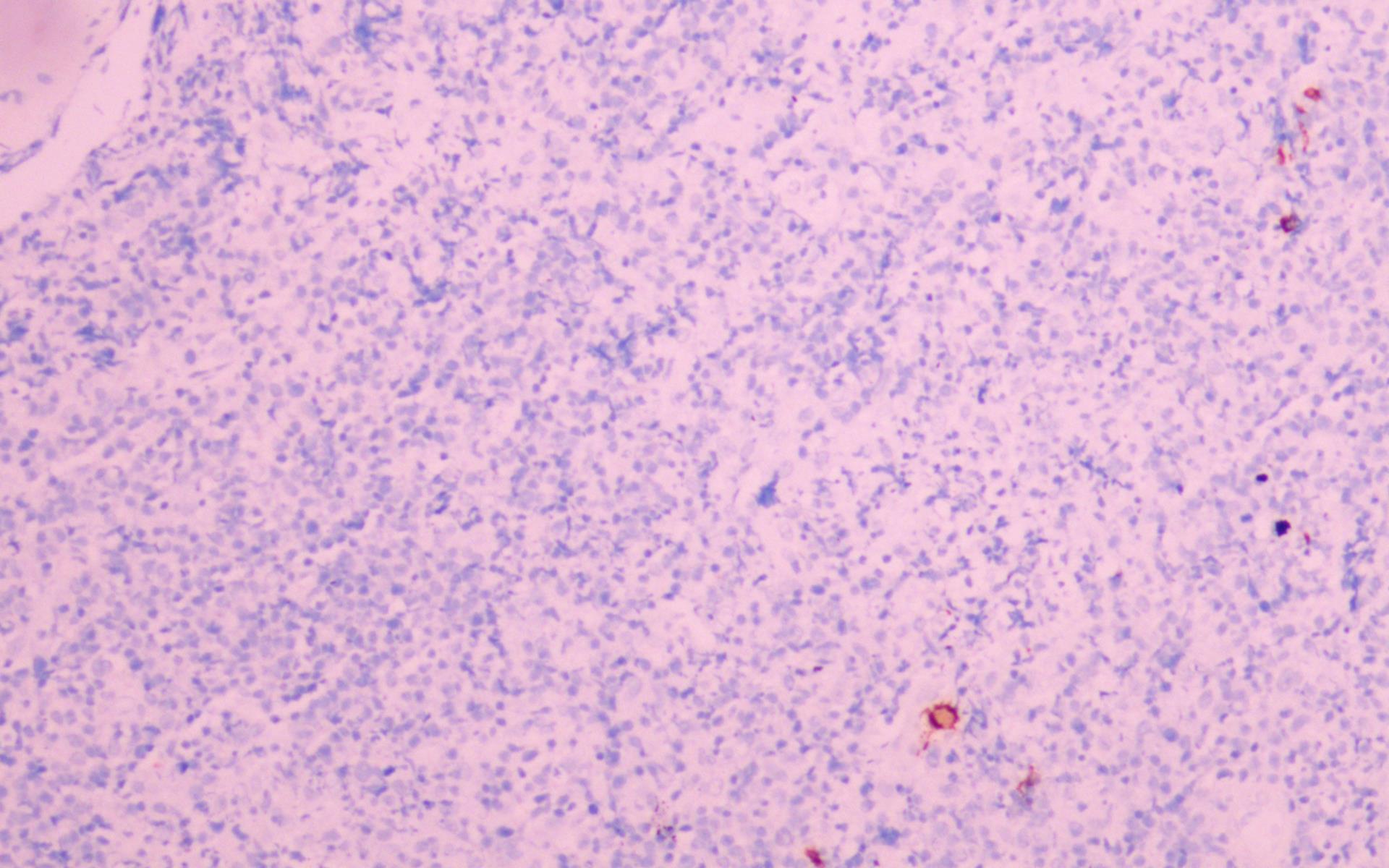


Ki-67 ×100
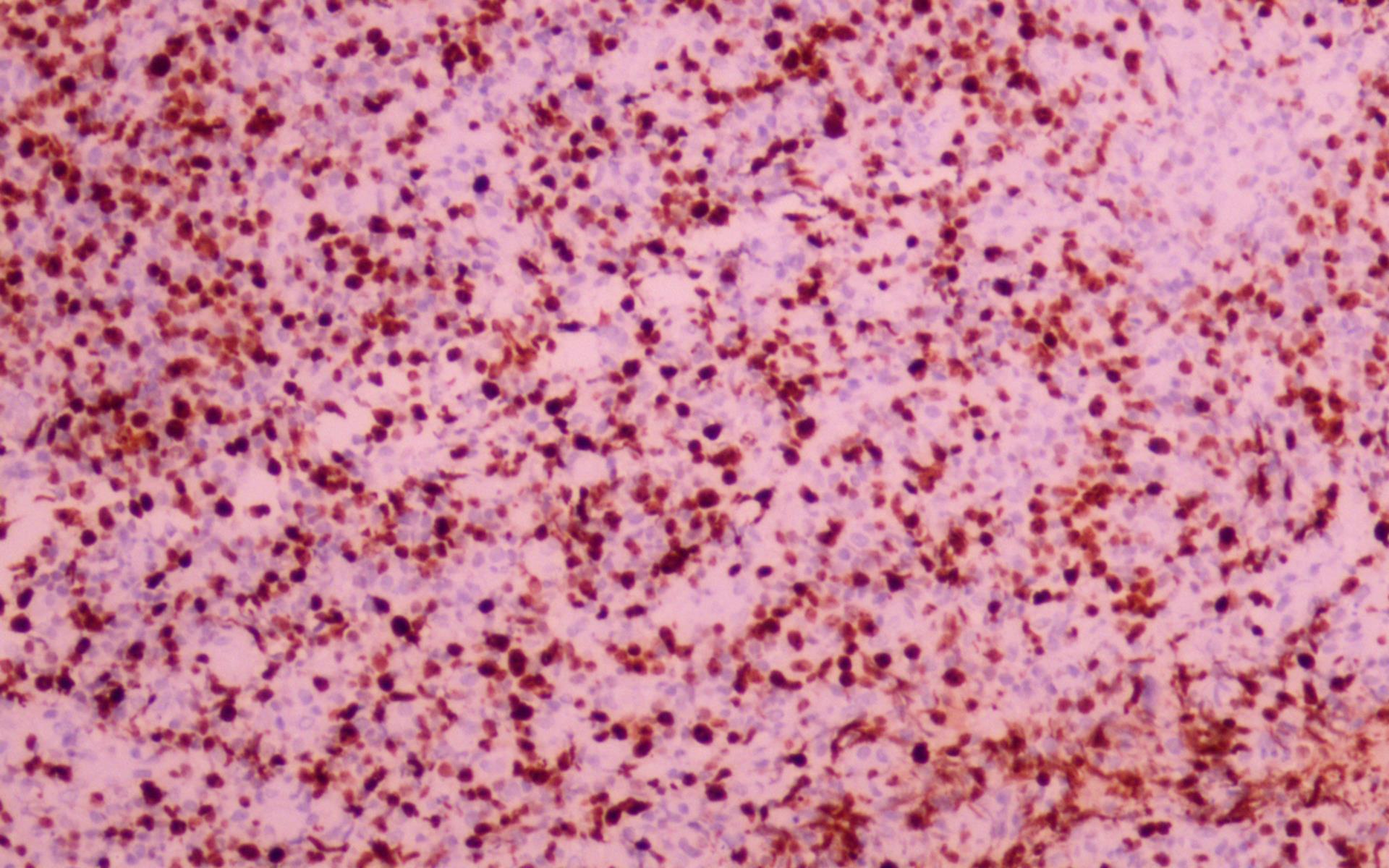
MUM1 ×100
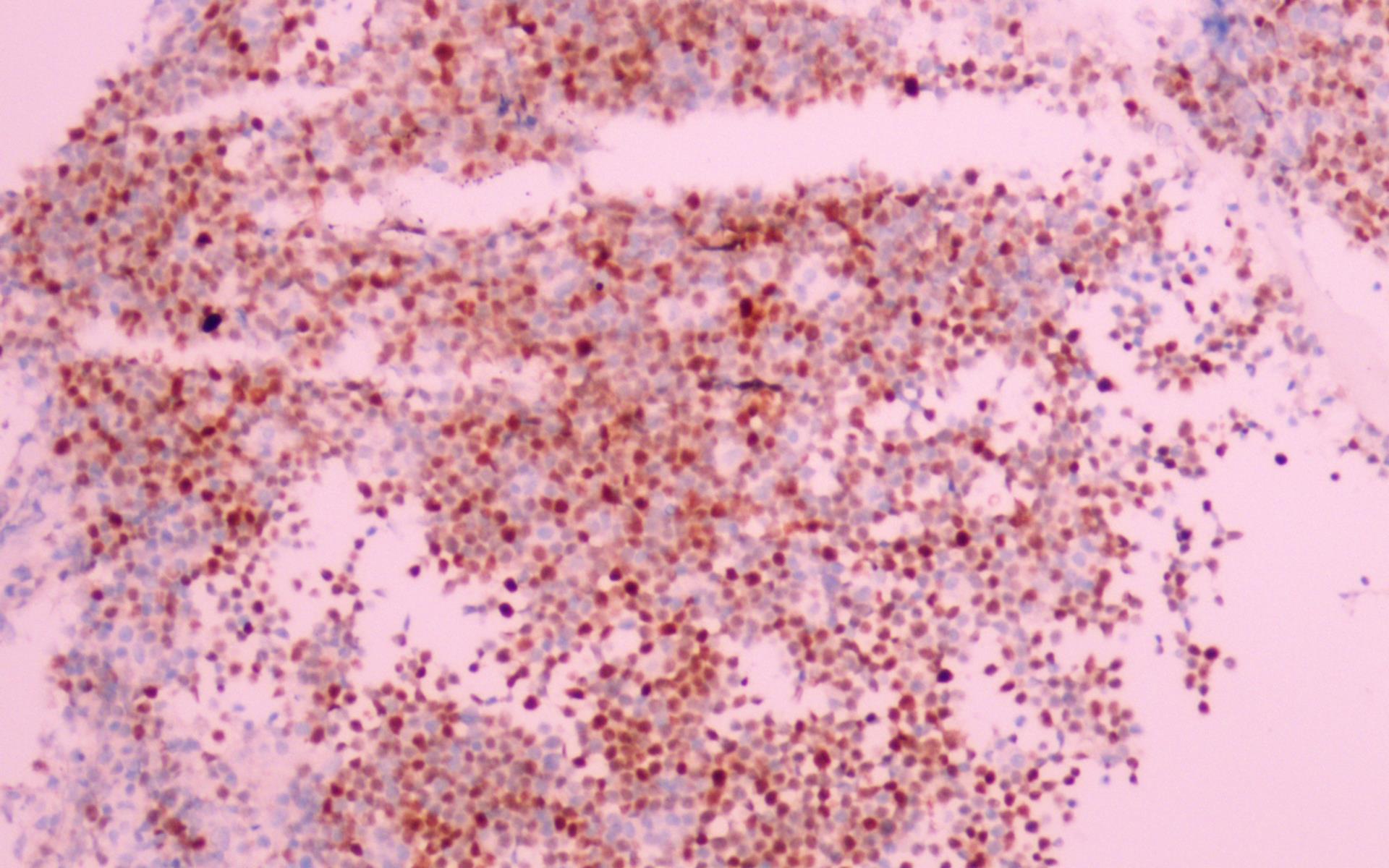


P53 ×100
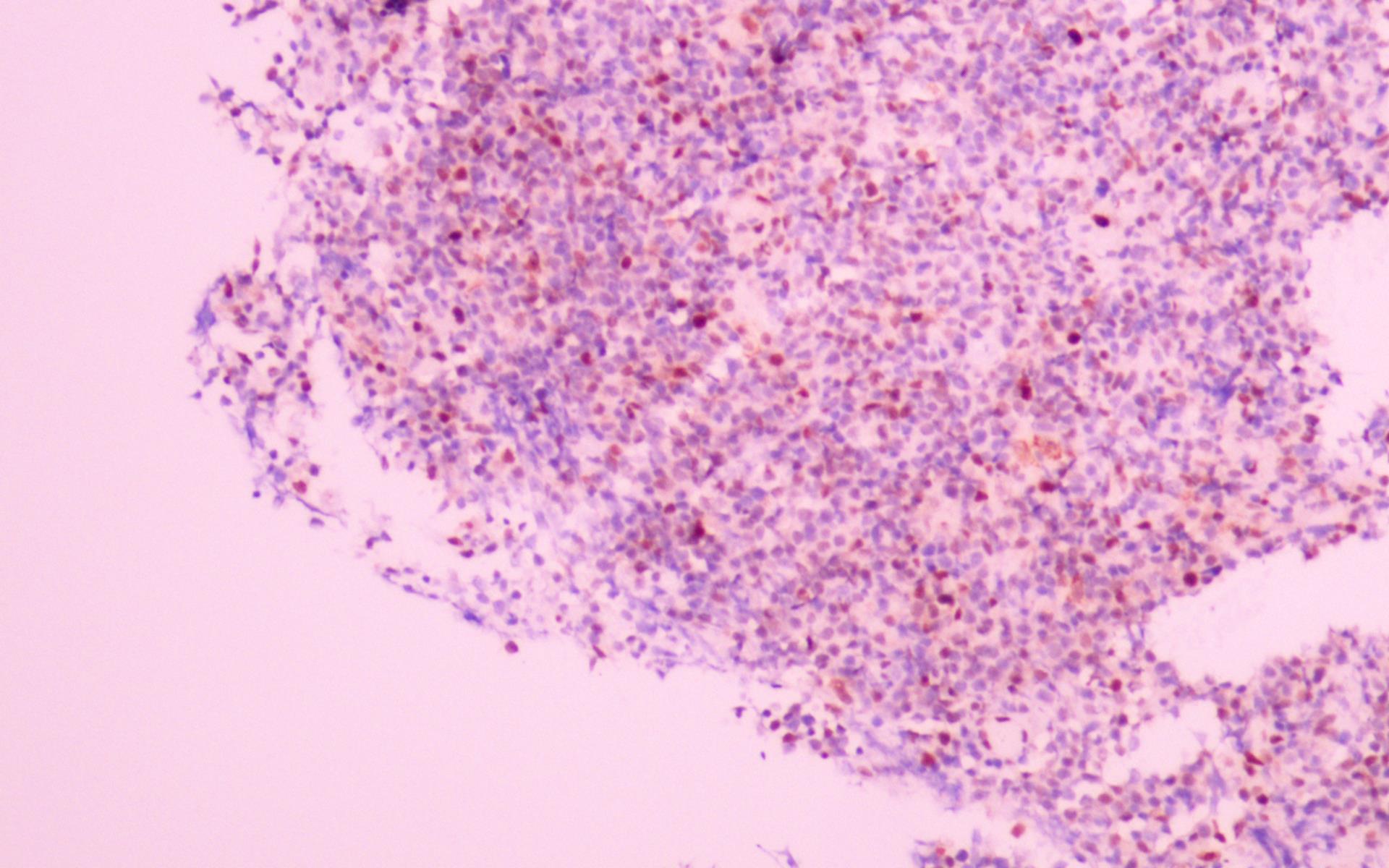
PAX5 ×100
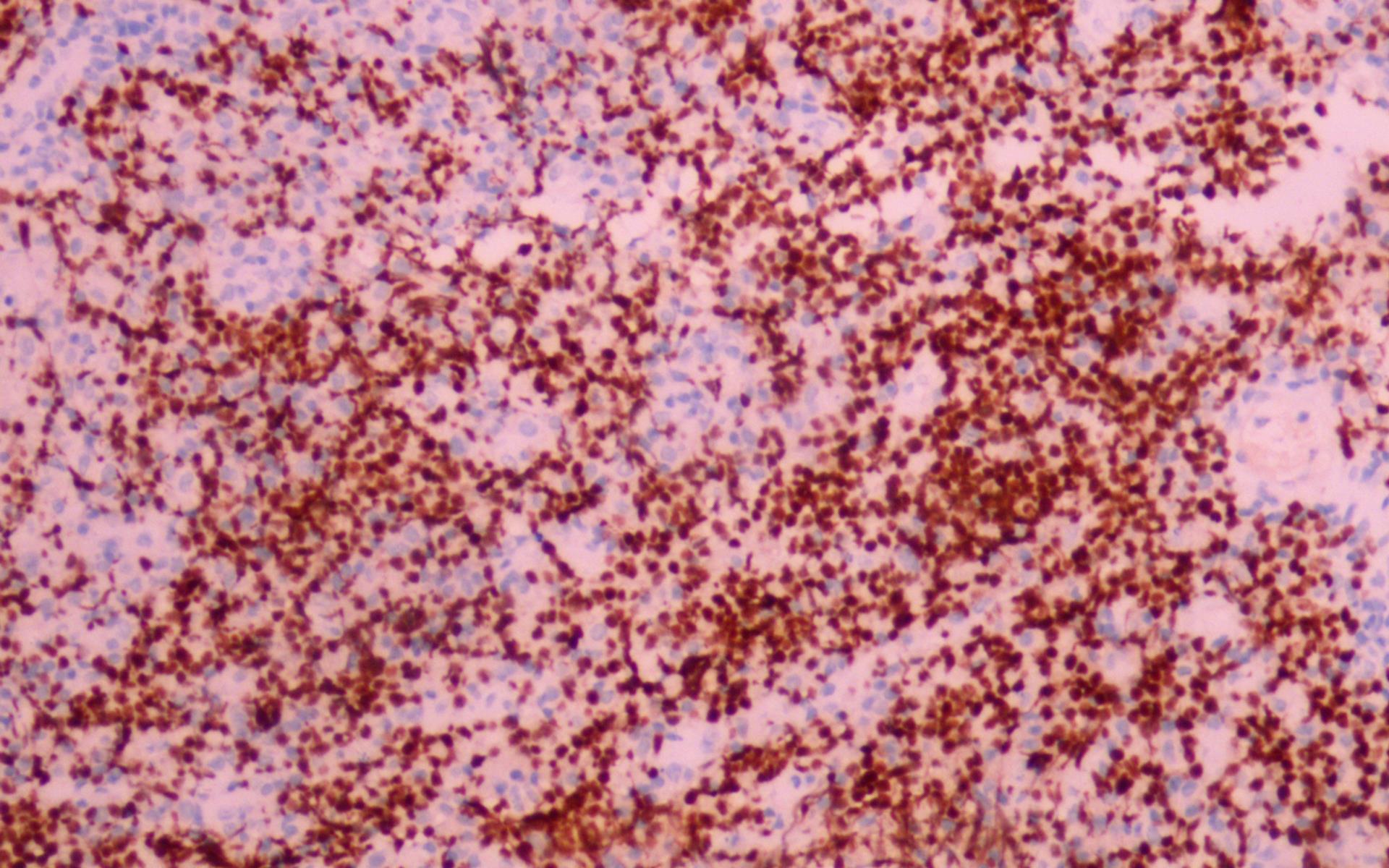


Figure 2: Immunohistochemical results of the temporal muscle lesion

bcl6 ×100


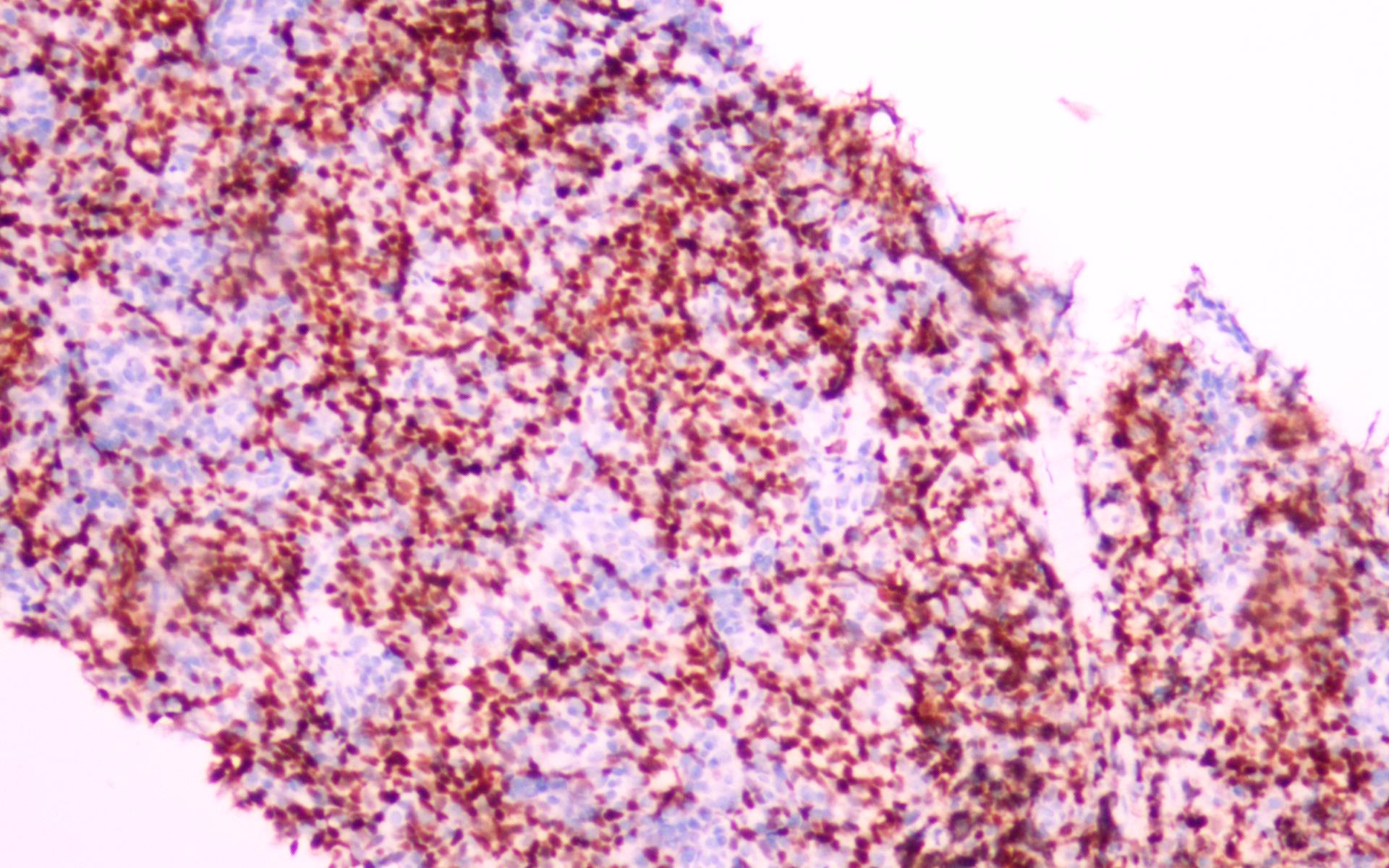
CD3 ×100
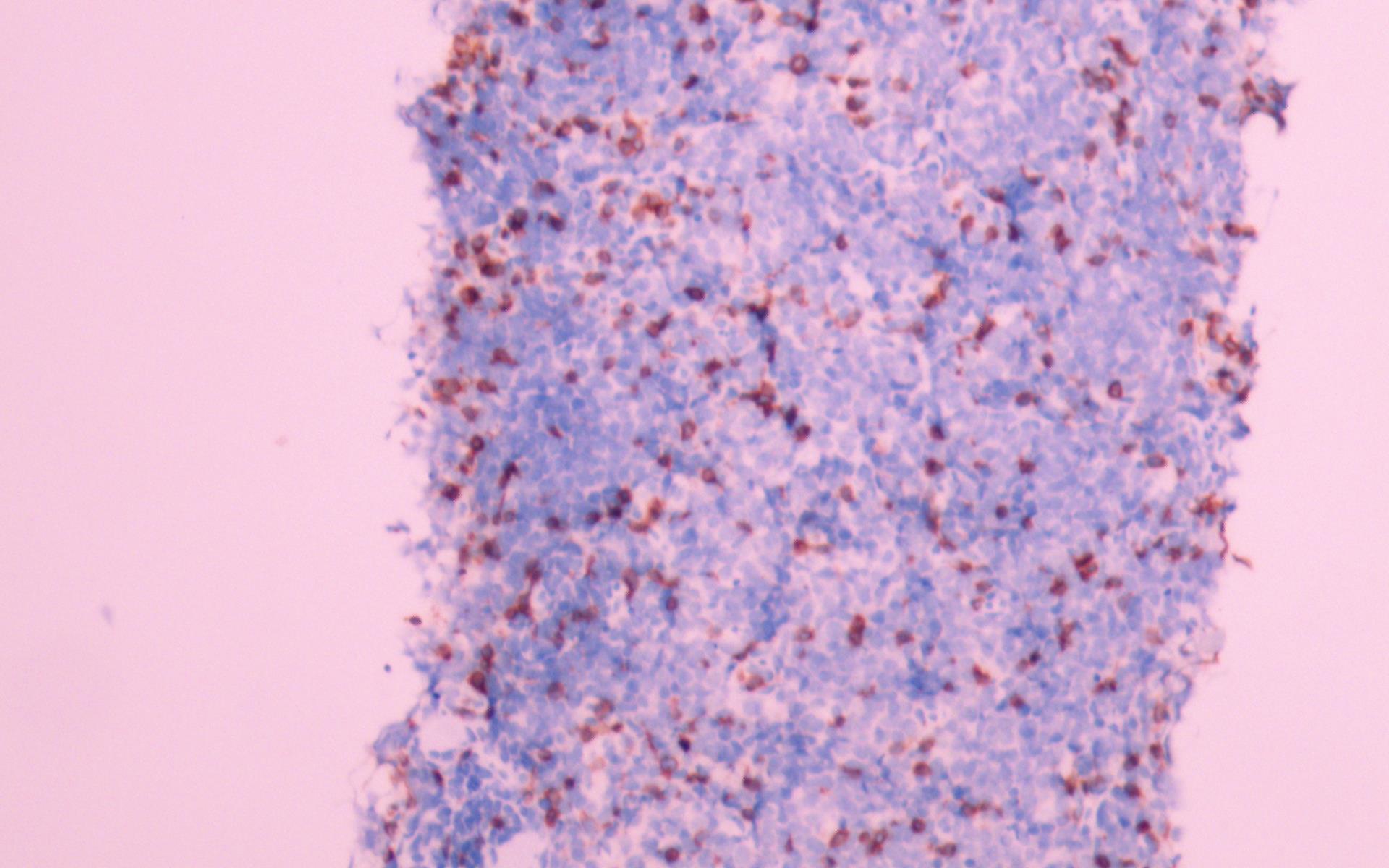


CD10 ×100
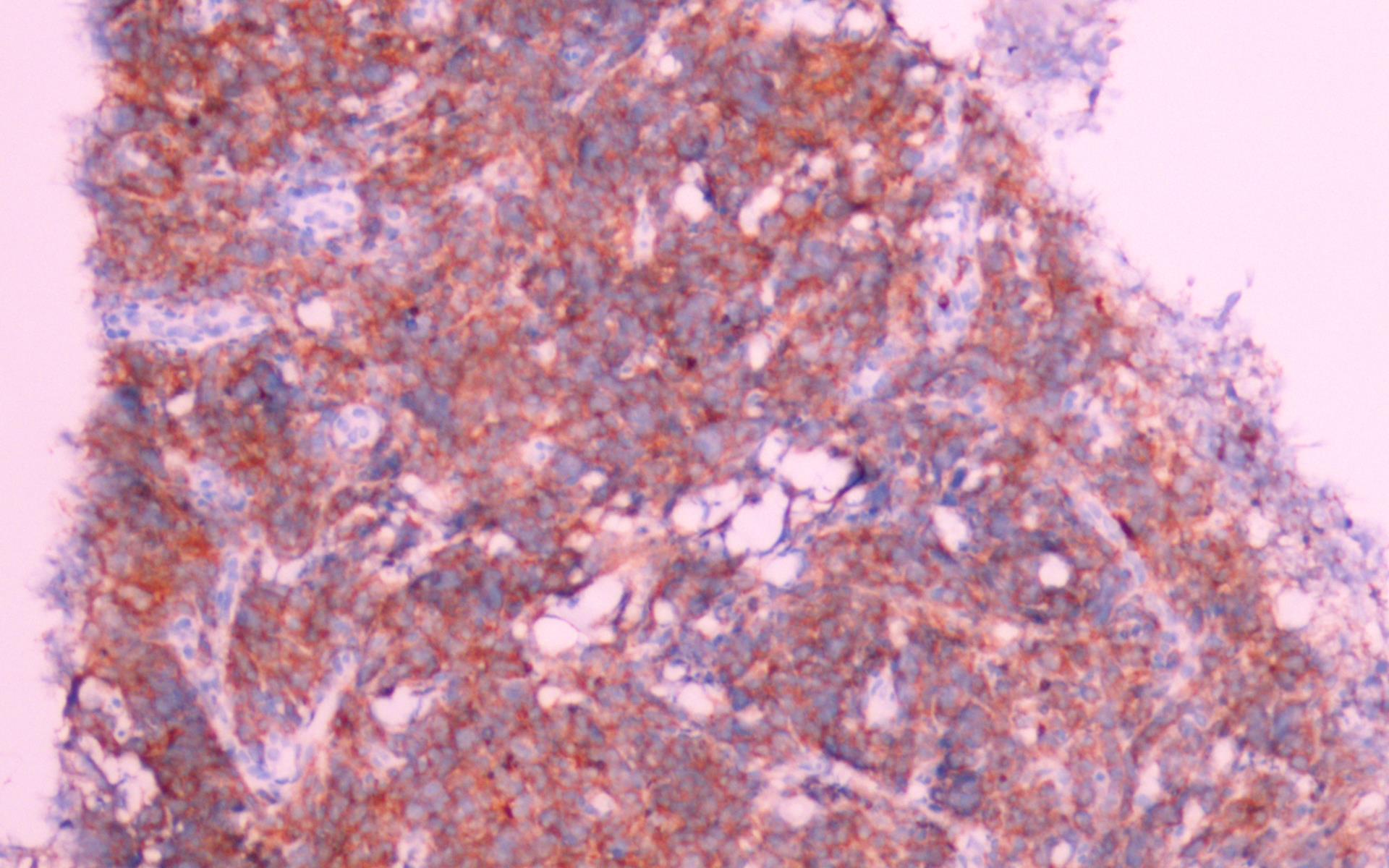
CD20 ×100
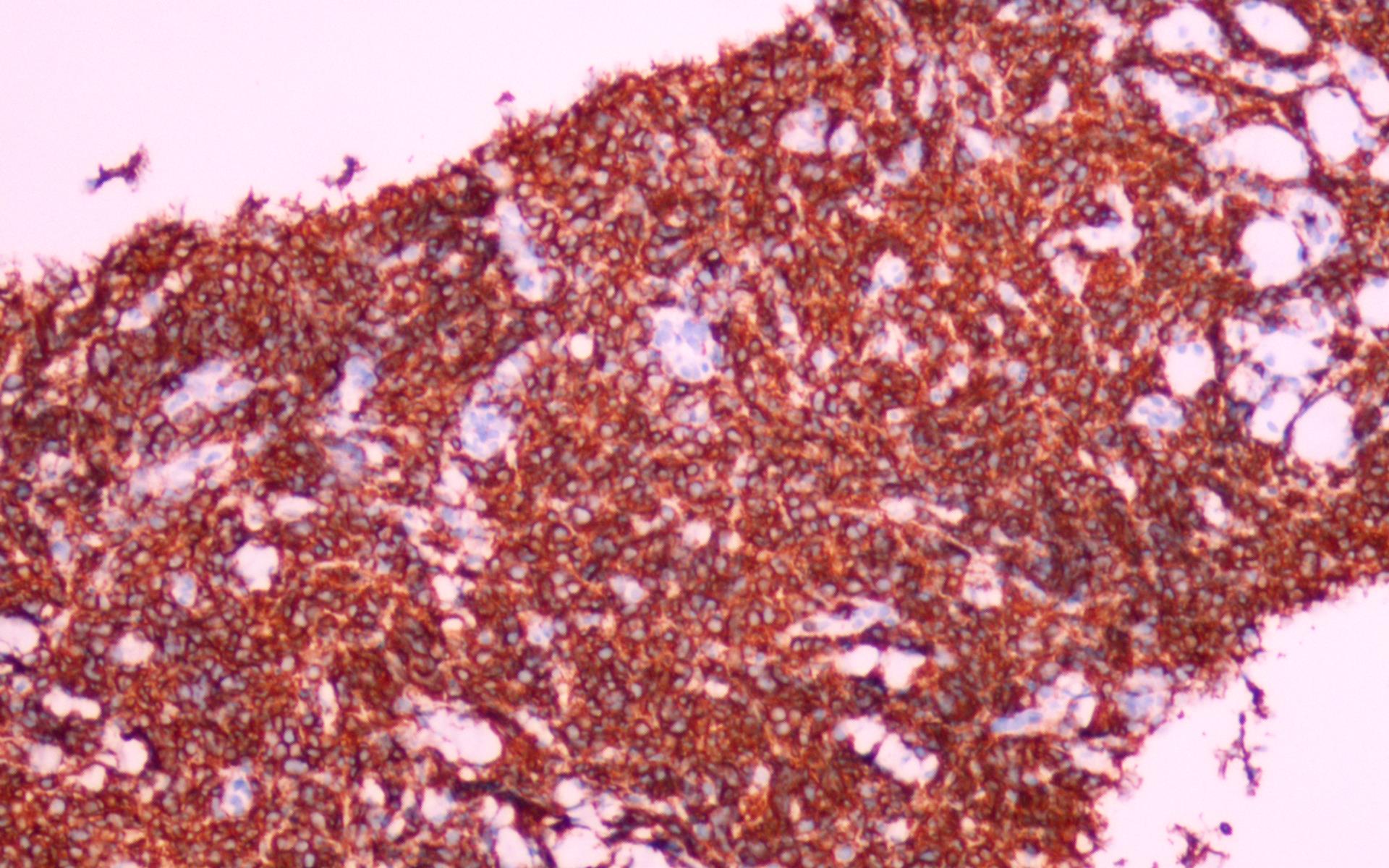


CD21 ×100
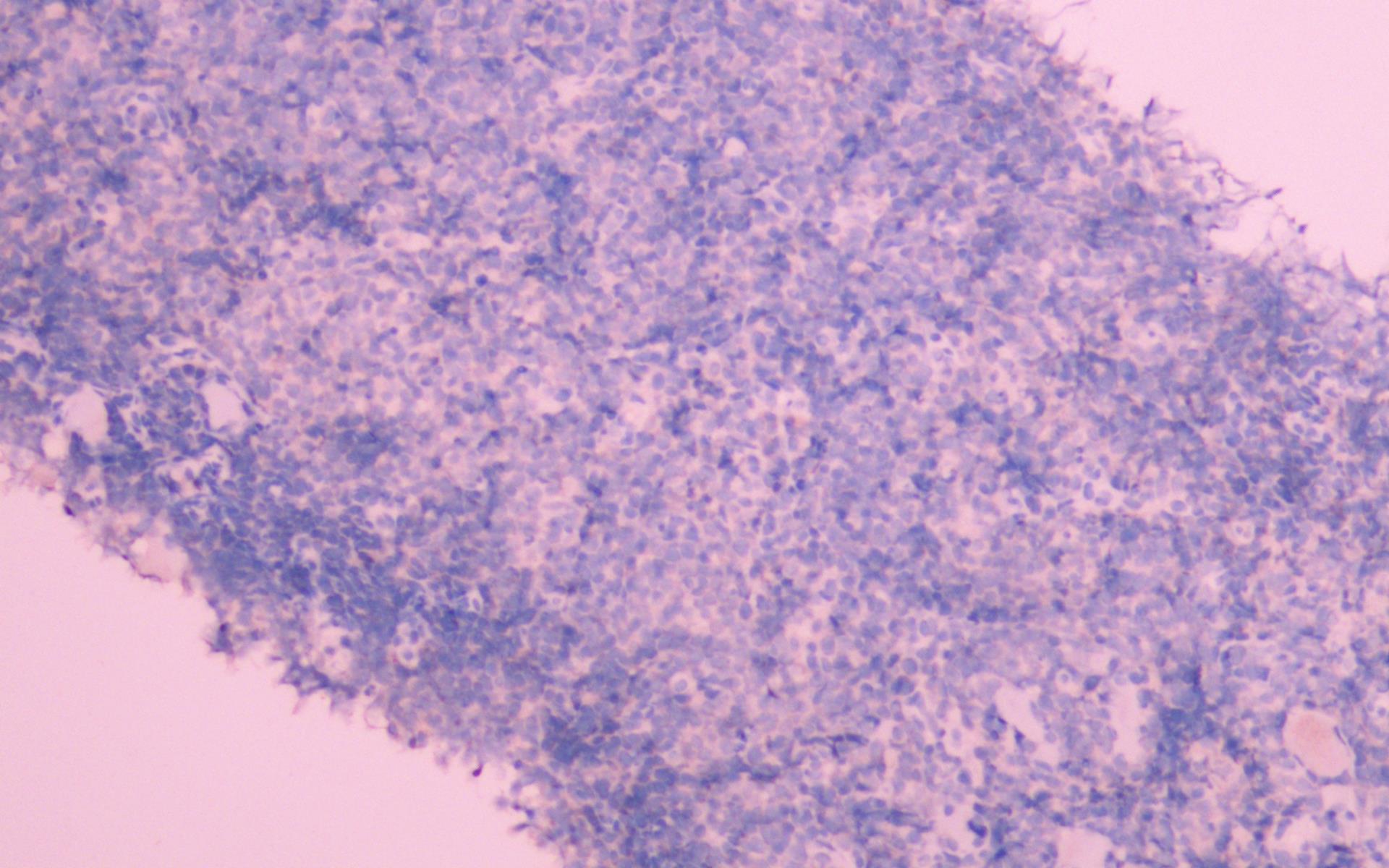
cMYC ×100
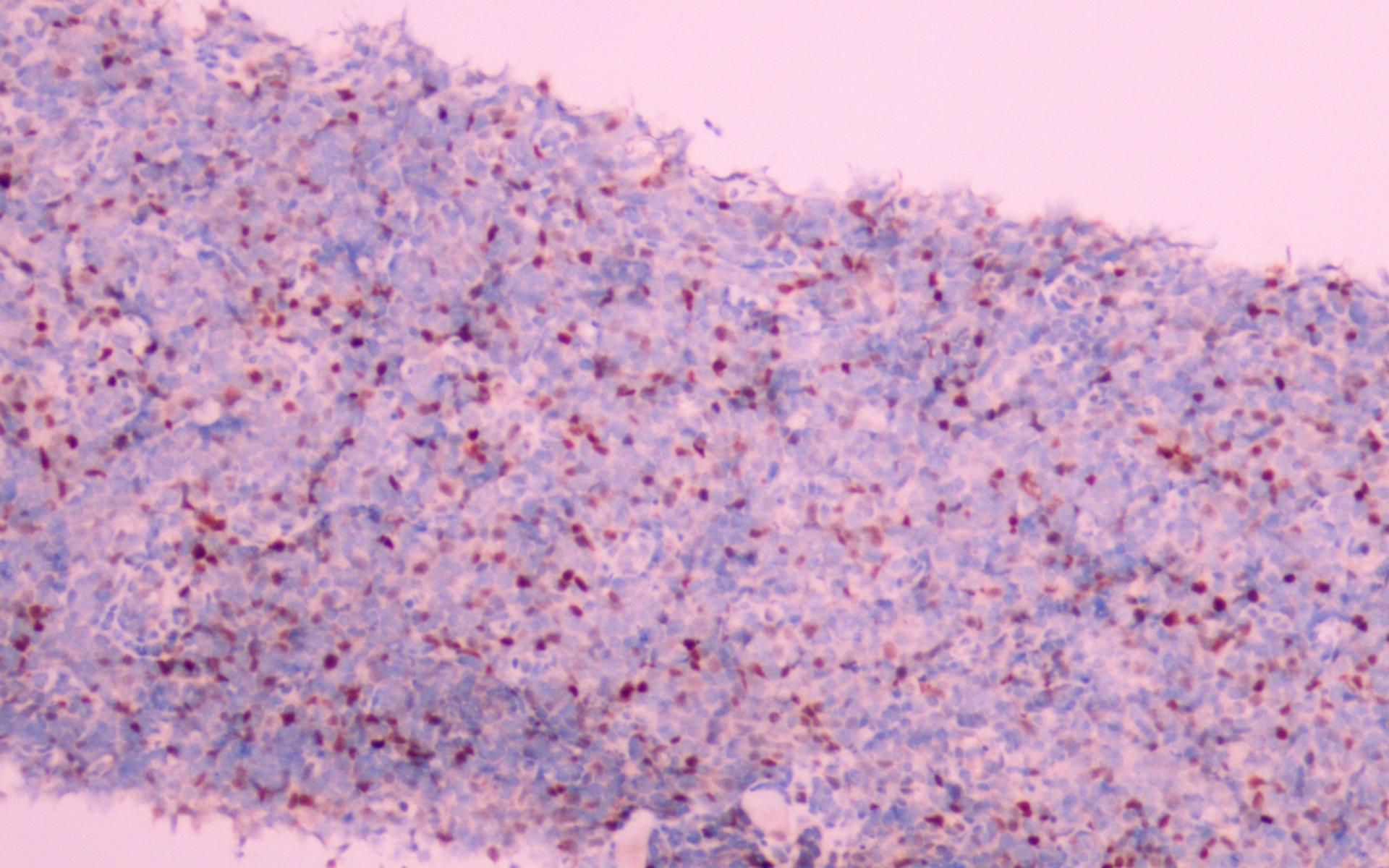


KI67 ×100
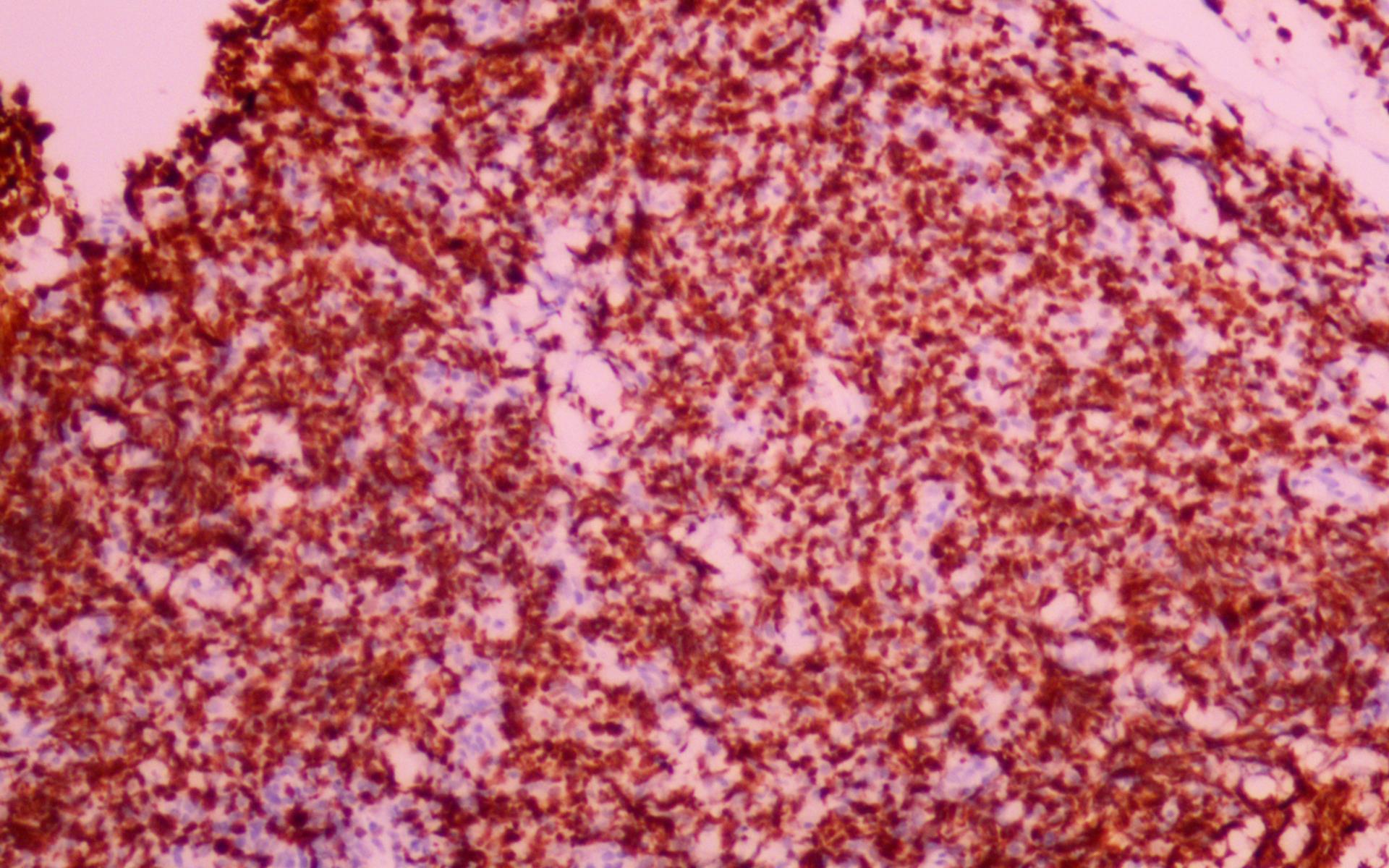
MUM1 ×100
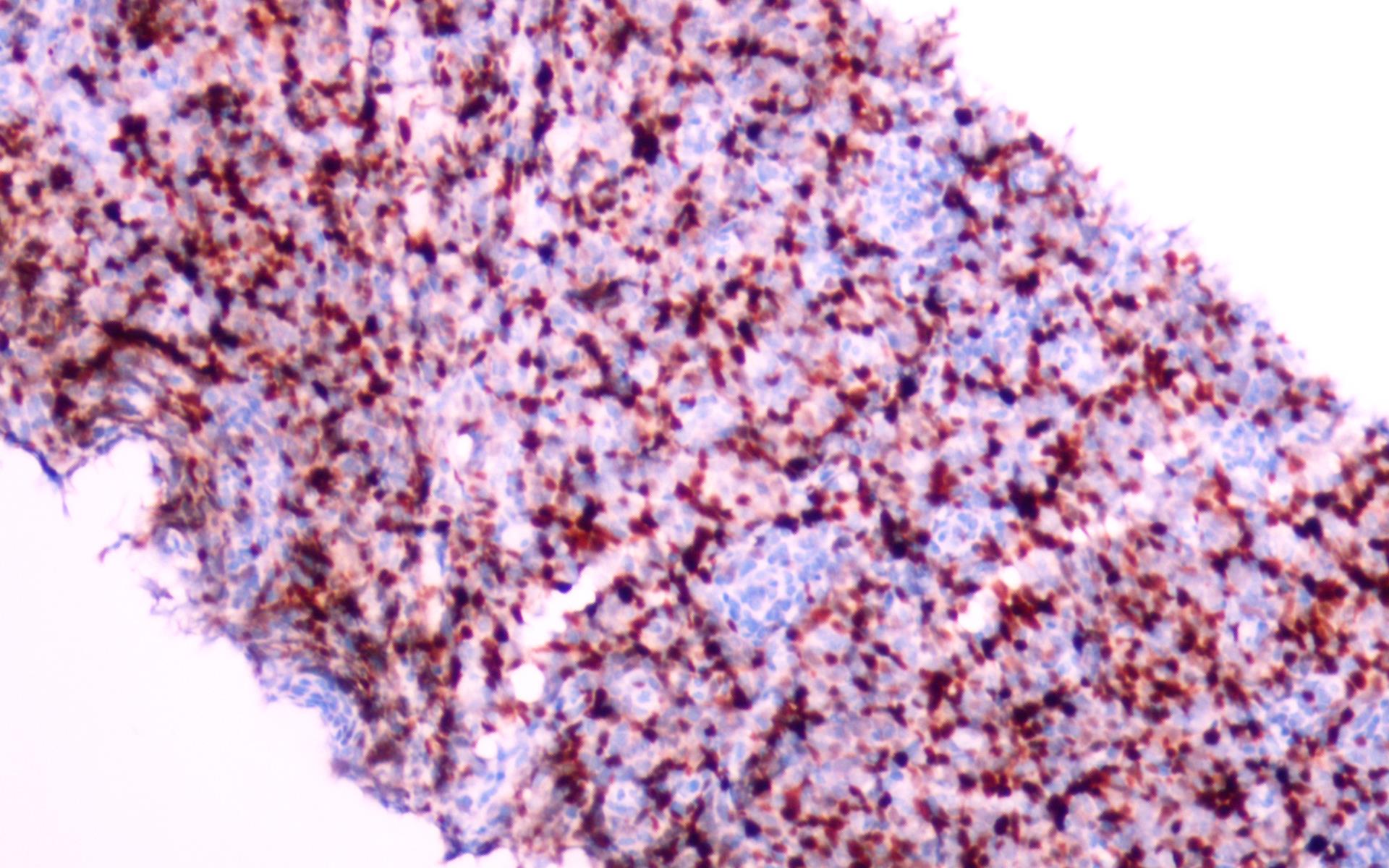


P53 ×100
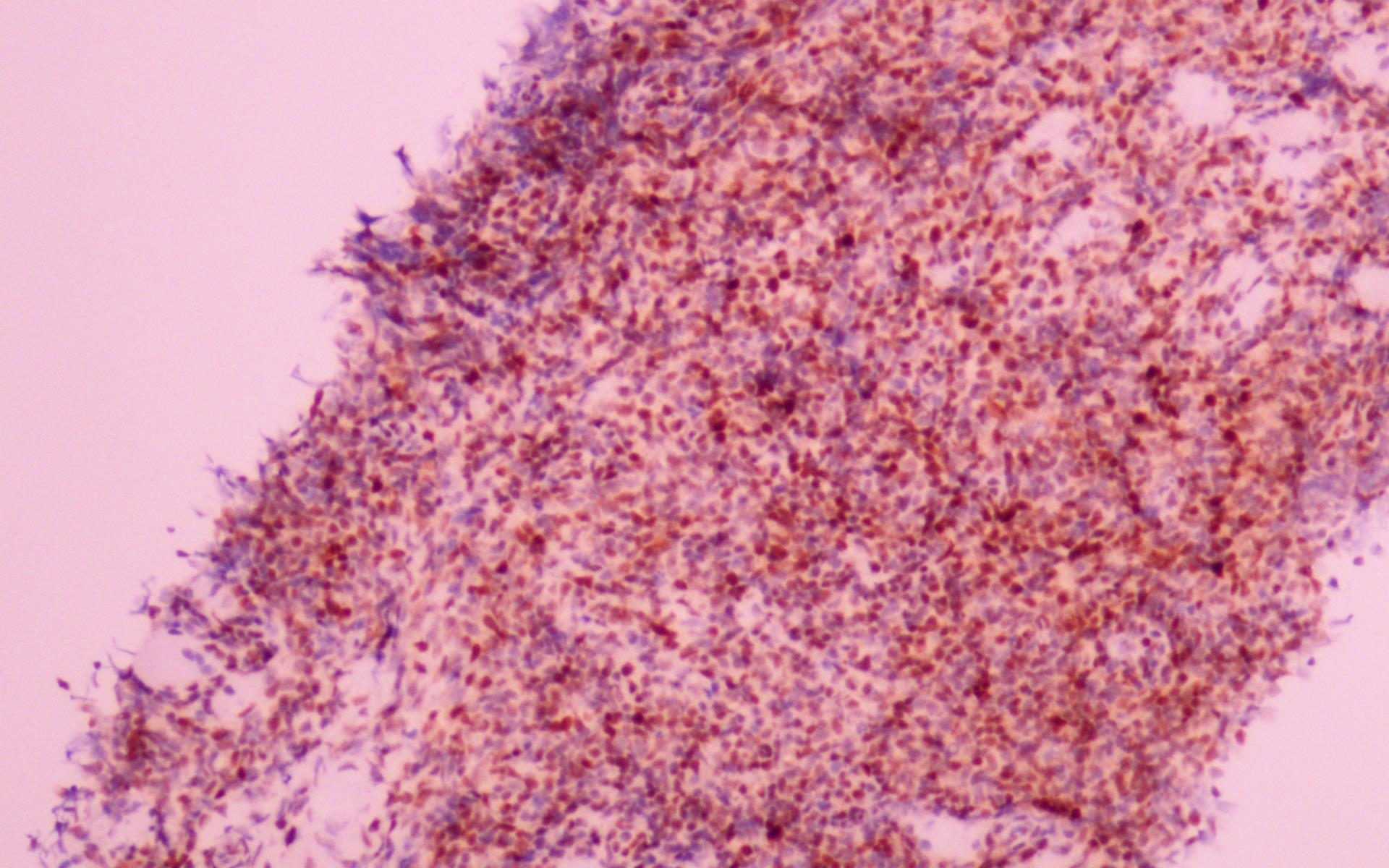

Supplement: Supplementary file 1 [file Table1.docx]
